# Exploration of heterogeneity and recurrence signatures in hepatocellular carcinoma

**DOI:** 10.1002/1878-0261.70012

**Published:** 2025-02-28

**Authors:** Wen‐Jing Wu, Jianchao Wang, Fuqing Chen, Xuefeng Wang, Bin Lan, Ruyi Fu, Hong Wen, Fangfang Chen, Wei Hong, Tian‐Yu Tang, Ying He, Gang Chen, Jianyin Zhou, Hai‐Long Piao, Di Chen, Shu‐Yong Lin

**Affiliations:** ^1^ State Key Laboratory of Cellular Stress Biology, Faculty of Medicine and Life Sciences, School of Life Sciences Xiamen University Xiamen China; ^2^ Department of Pathology, Clinical Oncology School of Fujian Medical University Fujian Cancer Hospital Fuzhou China; ^3^ Department of Hepatobiliary Surgery, Xiamen Key Laboratory of Translational Medical of Digestive System Tumor, Fujian Provincial Key Laboratory of Chronic Liver Disease and Hepatocellular Carcinoma, Zhongshan Hospital of Xiamen University, School of Medicine Xiamen University Xiamen China; ^4^ Laboratory of Radiation Oncology and Radiobiology, Innovation Center for Cancer Research Clinical Oncology School of Fujian Medical University and Fujian Cancer Hospital Fuzhou China; ^5^ Faculty of Health Sciences and Sports Macao Polytechnic University Macao China; ^6^ Laboratory Animal Center Xiamen University Xiamen China; ^7^ State Key Laboratory of Phytochemistry and Natural Medicines, Dalian Institute of Chemical Physics Chinese Academy of Sciences Dalian China; ^8^ Department of Digestive Diseases, Faculty of Medicine and Life Sciences, School of Medicine Xiamen University Xiamen China

**Keywords:** early‐relapse hepatocellular carcinoma, heterogeneity, machine learning, prognostic risk model, single‐cell RNA sequencing

## Abstract

Hepatocellular carcinoma (HCC), the sixth most prevalent cancer globally, is characterized by high recurrence rates and poor prognosis. Investigating the heterogeneity of relapsed HCC and identifying key therapeutic targets may facilitate the design of effective anticancer therapies. In this study, integrative analysis of single‐cell RNA sequencing data of primary and early‐relapsed HCC revealed increased proportions of infiltrating CD8^+^ T cells along with malignant cells and a decrease in CD4^+^ T cells in relapsed HCC. Cellular interaction and immunohistochemical analysis proposed MIF‐(CD74 + CXCR4) signaling pathway as a key mechanism by which malignant cells influence immune cells within the tumor microenvironment. Notably, primary malignant cells showed greater differentiation and proliferation potential, whereas relapsed cells exhibited enhanced epithelial–mesenchymal transition and inflammation, along with upregulated glycogen synthesis and metabolism‐related gene expression. Using machine learning techniques on bulk RNA‐seq data, we developed a relapsed tumor cell‐related risk score (RTRS) that independently predicts overall and recurrence‐free survival time with higher accuracy compared with conventional clinical variables. Prognostic biomarkers and potential therapeutic targets were validated via RT‐qPCR using mouse implantation models. This comprehensive investigation elucidates the heterogeneity of relapsed HCC and constructs a novel postoperative recurrence prognostic model, paving the way for targeted therapies and improved patient outcomes.

## Graphical abstract

This study leveraged public datasets and integrative bioinformatic analysis to dissect malignant cell heterogeneity between relapsed and primary HCC, focusing on intercellular communication, differentiation status, metabolic activity, and transcriptomic profiles. We developed a relapsed tumor cell‐related risk score (RTRS) model with prognostic relevance. Our findings highlight the utility of computational approaches in advancing precision oncology strategies for recurrent HCC.

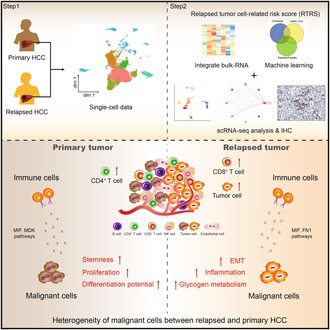

**Abbreviations**
GEO: Gene Expression Omnibus
HCC: hepatocellular carcinoma
IHC: immunohistochemistry
OS: overall survival
PCA: principal component analysis
PT: primary tumors
RFS: recurrence‐free survival
RT: relapsed tumors
RTRS: relapsed tumor cell‐related risk score
scRNA‐seq: single‐cell RNA sequencing
TCGA: The Cancer Genome Atlas
TSNE: T‐distributed stochastic neighbor embedding
UMAP: the uniform manifold approximation and projection

## 1 Introduction

Hepatocellular carcinoma stands as one of the most prevalent and deadliest malignancies, emerging as a primary contributor to cancer‐related mortality [1, 2]. Surgical treatment offers the best opportunity for long‐term survival in patients with HCC, primarily including hepatectomy and liver transplantation [3]. However, the high recurrence rate of tumors has become the greatest obstacle to the effectiveness of surgical treatment [3, 4]. Follow‐up studies of postoperative patients have shown that the 5‐year cumulative recurrence rate can reach as high as 70–80% [4]. Among them, early recurrence within 2 years after surgery accounted for 70% of relapsed HCC cases and was difficult to cure and significantly associated with a very poor prognosis [5, 6]. Therefore, reducing postoperative recurrence rates is crucial for improving patient survival and quality of life [7]. The heterogeneity and diversity of liver cancer are key factors contributing to the elevated postoperative recurrence rates [8, 9]. Furthermore, the perturbation and alteration of the tumor microenvironment induced by surgery may promote the growth of residual liver cancer cells and consequently contribute to tumor recurrence [4]. Currently, research on the molecular mechanisms of HCC predominantly focuses on primary tumors, while molecular mechanisms associated with postoperative tumor recurrence remain largely elusive. Therefore, investigating the molecular mechanisms of early‐relapse HCC presents a significant challenge, underscoring the urgent need for the development of novel biomarkers to effectively prevent postoperative recurrence in patients with HCC. This will contribute to improving their survival prognosis.

Tumors are a complex system composed of various cell types (including malignant cells, immune cells, and stromal cells) as well as noncellular matrix components. Furthermore, there exist temporal and spatial interactions among these cells [10, 11]. The complex tumor microenvironment profoundly undermines the effectiveness of immunotherapy [12]. Tumor heterogeneity, closely intertwined with clinical diagnosis and treatment, represents a formidable challenge in realizing precision medicine and overcoming cancer. Hence, it is crucial to explore the spatiotemporal interactions among cells and the intricate mechanisms of the microenvironment in tumor tissue at the single‐cell level. Single‐cell sequencing technology serves as a cutting‐edge tool for investigating specific tumor subtypes, the complex components of the tumor microenvironment, and intercellular interactions [13, 14, 15]. Currently, significant progress has been made in studying the heterogeneity and immune cell subpopulations of primary HCC using single‐cell sequencing technology [16, 17, 18]. Though a limited number of studies have also explored relapsed HCC at the single‐cell level [5, 19], there remains a notable deficiency in comprehensive and in‐depth research into the mechanisms of HCC recurrence. Additionally, suitable biomarkers for predicting recurrence‐free survival (RFS) and preventing postoperative recurrence of HCC have not yet been developed. Machine learning, a key component of artificial intelligence, can be used to identify and understand patterns within the overall information volume in order to construct classification and prediction models [20]. The application of machine learning in the medical field, especially in tumor research, is increasingly prevalent [21, 22].

In this study, we comprehensively investigated the characteristics of cell interactions and the heterogeneity of malignant cells in various aspects, such as metabolism and transcription, in relapsed HCC by integrating multi‐omics analyses along with immunohistochemistry (IHC) and RT‐qPCR. Additionally, we developed an effective RTRS model for predicting prognosis using multiple machine learning methods. This research offers valuable insights into the mechanisms underlying HCC recurrence and provides a reference for achieving precision medicine in patients with HCC.

## 2 Materials and methods

### 2.1 Patient samples

Unstained paraffin‐embedded 5 mm tissue sections of 12 HCC samples were obtained from Fujian Medical University Cancer Hospital (Fujian, China) (between January 2021 and June 2022). All procedures were conducted in accordance with the Declaration of Helsinki and with approval from the ethics committee of Fujian Cancer Hospital (ethics number: K2023‐412‐01). Written informed consent was obtained from all participants. Detailed clinical and pathological information of patients is shown in Table S1.

### 2.2 Cell culture

Hepa1‐6 cells (RRID: CVCL_0327), obtained from the American Type Culture Collection (ATCC, Manassas, VA, USA), were cultured in DMEM supplemented with 10% FBS, 100 IU penicillin, and 100 μg·mL^−1^ streptomycin. Cells were maintained at 37 °C in a humidified incubator containing 5% CO_2_. The cell line was screened for mycoplasma contamination and authenticated within the past 3 years using STR profiling.

### 2.3 Animal and HCC xenografts

Male C57BL/6J mice (7–8 weeks old, 18–20 g) were purchased from Xiamen University Experimental Animal Center and housed in a specific pathogen‐free environment with controlled temperature (22–24 °C), humidity (55–60%), and a 12 h light/dark cycle (lights on from 08:00 to 20:00). Mice received *ad libitum* access to water and a standard chow diet (65% carbohydrate, 11% fat, 24% protein). All animal experiments were approved by the Animal Care and Use Committee of Xiamen University (ethics approval No.: XMULAC20240101). For orthotopic tumor implantation, Hepa1‐6 cells were detached at 80–90% confluence using 0.25% trypsin–EDTA, washed twice in serum‐free DMEM medium, and resuspended to 5 × 10^7^ cells·mL^−1^. For surgical implantation, mice were anesthetized with 2,2,2‐tribromoethanol (250 mg·kg^−1^; T48402‐100G; Sigma, Saint Louis, MI, USA), and a 1 cm incision was made on the abdominal wall to expose the left hepatic lobe. A 29‐gauge syringe was used to inject 40 μL of cell suspension (2 × 10^6^ cells) beneath the hepatic capsule over 1 min. The injection site was immediately compressed with a sterile cotton‐tipped applicator for 2 min to ensure hemostasis and prevent cell reflux. The wound was subsequently sutured with 6–0 absorbable sutures, and the mouse was closely monitored until it fully recovered from anesthesia. All mice survived the procedure without acute complications. At the predefined experimental endpoint (Day 12 postimplantation), the mice were euthanized by CO_2_ inhalation, and the livers were excised. Each tumor‐bearing liver was systematically processed as follows. Representative tumor and adjacent fragments were immediately snap‐frozen in liquid nitrogen and stored at −80 °C for subsequent RNA extraction. The remaining tumor mass with adjacent hepatic parenchyma was fixed in 4% PFA (24 h, 4 °C) for paraffin embedding and sectioned for hematoxylin and eosin (H&E) staining. Histopathological assessment confirmed successful tumor engraftment, with a maximum tumor diameter below 15 mm in all mice.

### 2.4 Data source and preprocessing

The HCC single‐cell data were downloaded from the China National GeneBank DataBase (https://db.cngb.org) (CNSA: CNP0000650), including 12 primary HCC samples (P08, P09, P10, P11, P12, P13, P14, P15, P16, P17, P18, and P19) and six early‐relapsed HCC samples (P01, P02, P03, P04, P05, and P07). The 18 HCC samples were transformed into a Seurat object using the ‘CreateSeuratObject’ function from the seurat package (v4.4.0) [23], filtering for cells with 200–10 000 features and genes expressed in at least 10 cells. Moreover, cells with mitochondrial content exceeding 20% were excluded.

Bulk RNA sequencing data and related clinical information of 371 patients with HCC were downloaded from the TCGA (https://cancergenome.nih.gov/) database and Xena Functional Genomics Explorer (https://xenabrowser.net/) as a training cohort. Next, The GSE14520, including 242 patients with HCC as a validation cohort, was downloaded from the Gene Expression Omnibus (GEO) (https://www.ncbi.nlm.nih.gov/geo/) database. If the patient relapses during postoperative follow‐up, its status is categorized as ‘Recurred’, otherwise it is defined as ‘Recurrence Free’ The duration from operation to disease recurrence or the last follow‐up was termed as RFS time. Additionally, we excluded HCC samples with an overall survival (OS) time of less than 30 days for further analysis after preprocessing.

### 2.5 Dimension reduction and clustering analysis of scRNA sequencing data

The gene expression matrices were generated by log normalization and linear regression using the SCTransform method with 3000 highly variable features. After principal component analysis (PCA), the T‐distributed stochastic neighbor embedding (TSNE) and the uniform manifold approximation and projection (UMAP) were used to visualize the cell clusters identified by the ‘FindNeighbors’ and ‘FindClusters’ functions. We integrated all cells by the harmony algorithm in the harmony r package (v1.0.3) [24] to eliminate the batch effect before clustering analysis. Finally, the cell types were initially identified using the singler software (v2.2.0) [25], followed by further identification of cell types through specific marker genes of cell subpopulations. Differentially expressed genes (DEGs) in each cell population were identified by the ‘FindAllMarkers’ function (min.pct = 0.25, logfc.threshold = 0.25, *P* < 0.05).

### 2.6 Identification of malignant cells

To identify potential malignant cells, we utilized the infercnv r package (v1.19.1) [26] to infer the chromosomal copy number variations of each cell. Two analytical approaches were employed for inference: all immune cells as the reference group or without a reference group. Other parameters were set to default. Ultimately, we defined cells with clonal large‐scale chromosome copy number variations as malignant.

### 2.7 Cell–cell communication analysis

We inferred and visualized cell–cell interaction networks involving ligand‐receptor pairs based on the gene expression information inherent in the scRNA‐seq data using the cellchat (v1.6.1) r package [27, 28], with default parameters set. The list of known ligand‐receptor pairs was derived from CellChatDB [29], which serves as a repository for novel ligands, receptors, and their interactions. We visualized the differential number of interactions or interaction strength among different cell populations using the ‘netVisual_circle’ function. Additionally, we utilized the ‘netVisual_bubble’ function to display all significant signaling pathways from tumor cells to other major immune cells.

### 2.8 Identification of tumor cell subpopulation features in RT and PT

We extracted UMI raw counts of tumor cells from the complete dataset. The raw counts were normalized, and variable genes were identified by the ‘NormalizeData’ and ‘FindVariableFeatures’ functions in Seurat, respectively. We integrated all cells by the r package harmony [24] to re‐eliminate the batch effect before clustering analysis. Tumor cell clusters were identified using SNN‐based clustering based on the first 15 principal components with resolution = 0.05. The epithelial–mesenchymal transition (EMT) scores were calculated using the ‘AddModuleScore’ function, applying the ‘HALLMARK_EPITHELIAL_MESENCHYMAL_TRANSITION’ geneset from GSEA MSigDB (https://www.gsea‐msigdb.org/gsea/msigdb). To infer transcription factor regulon activity, regulon analysis was performed as described previously [30, 31], using pyscenic (v0.12.1). The cytotrace2 (v1.0.0) r package [32] was used to predict the differentiation scores of tumor cell clusters and to facilitate the construction of the pseudotime trajectory in monocle2. The pseudotime trajectory analysis of tumor cells was performed using monocle2 (v2.28.0) [33]. To investigate the metabolic heterogeneity between relapsed and primary tumor cells, we utilized the single‐cell Flux Estimation Analysis (scFEA) method [34] to evaluate the metabolic flux of tumor cells. The original expression matrix of tumor cells was used as the input file, and the default parameters were applied. Following normalization, differences in the activity of glycolysis and TCA cycle‐related metabolic pathways across various tumor cell subpopulations were visualized.

### 2.9 Differentially expressed gene analysis for RT and PT malignant cells

To define feature genes in malignant cells from RT, we performed differential expression analysis between malignant cells from RT and PT using ‘FindMarkers’ function of Seurat, with log‐scaled fold change ≥ 0.25 and adjusted *P* value < 0.05 (Wilcoxon rank‐sum test, adjusted by Bonferroni correction). Differentially expressed genes passing the criteria are shown in Table S2.

### 2.10 GO and pathway enrichment analyses

To explore the biological processes and pathways associated with DEGs, we performed Gene Set Enrichment Analysis (GSEA), Kyoto Encyclopedia of Genes and Genomes (KEGG) pathway enrichment analysis, and Gene Ontology (GO) enrichment analysis. This part of the analysis mainly used the clusterprofiler r package (v4.8.3) and the Metascape online site (https://metascape.org/gp/index.html#/main/step1).

To score individual cells for pathway activities, we used the r package aucell (v1.22.0). First, we used an expression matrix to compute the gene expression rankings in each cell by the AUCell_buildRankings function with default parameters. The canonical pathway gene sets downloaded from the Msigdb database (https://www.gsea‐msigdb.org/gsea/msigdb) were then used to score each cell. The area under the curve (AUC) values were computed using the AUCell_calcAUC function based on gene expression rankings.

### 2.11 Constructing co‐expression networks from single‐cell transcriptomic data using hdWGCNA

To identify co‐expression modules of genes specifically associated with malignant cells, we conducted high‐dimensional weighted gene co‐expression network analysis in single‐cell and spatial transcriptomic data using the hdwgcna (v0.2.26) r package [35, 36]. In short, highly similar cells are collapsed into ‘metacells’ to reduce data sparsity while preserving cellular heterogeneity and allowing modular design for separate network analysis within specific cell populations. First, we utilized the SetupForWGCNA function and the MetaspotsByGroups function to create a new slot in the Seurat object to store an hdWGCNA experiment and compute metaspots, respectively. Then, the following functions were sequentially performed with default parameters: ‘TestSoftPowers’, ‘ConstructNetwork’, ‘ModuleEigengenes’, ‘ModuleConnectivity’, ‘ModuleExprScore’, and ‘RunModuleUMAP’.

### 2.12 Machine learning‐based prognostic model construction

To construct a RTRS model, we collected two bulk RNA‐seq datasets, TCGA‐LIHC (training cohort) and GSE14520 (test cohort). The RNA‐seq raw read count from the TCGA‐LIHC was converted to transcripts per kilobase million (TPM) and further log‐2 transformed. First, we intersected the significantly related gene modules of malignant cells, malignant cell marker genes, and DEGs between malignant cells from RT and PT. Then, a univariate Cox regression analysis of these intersecting genes was performed to identify potential prognostic factors (*P* < 0.05). We further utilized three machine learning methods, including the Lasso‐Cox algorithm, CoxBoost algorithm, and Random Survival Forest (RSF) algorithm, to screen prognostic biomarkers, and took the intersection of the biomarkers obtained by the three methods as the final prognostic genes related to tumor recurrence. Each algorithm is validated using tenfold cross‐validation. Finally, a prognostic model was constructed based on these genes and their Lasso‐Cox regression coefficients. Patients in the training cohort were divided into high‐score and low‐score groups according to the median risk score. The timeroc r package (v0.4) was utilized to generate receiver operating characteristic (ROC) curves, and the predictive ability of the prognostic signatures was evaluated by the AUC value of the ROC curve. Furthermore, the same algorithm was applied to the GSE14520 dataset to validate the prognostic value of the prognostic model.

### 2.13 Immune infiltration analysis in the tumor microenvironment

In scRNA‐seq data, we first calculated the average expression level of each prognostic gene for each patient sample. Based on these prognostic genes and the same method used in TCGA‐LIHC, the patient samples are then divided into high‐ and low‐risk groups. Then, we visualized the differences in the proportions of various cell subpopulations between the high‐ and low‐risk groups. In addition, the Pearson correlation between the expressions of relapse‐related prognostic genes and the proportions of infiltrating immune cells was calculated and visualized using the pheatmap r package (v1.0.12). Cell type deconvolution of TCGA‐LIHC samples was conducted using the bayesprism [37] r package (v2.2.2), with HCC single‐cell data utilized as a reference for cell type annotation. We further investigated the relationship between immune cells, such as CD8^+^ T cells and B cells, and survival rates in the TCGA‐LIHC using the survival r package (v3.7.0). Finally, we analyzed the differential expression of immune checkpoint‐related genes between high‐risk and low‐risk populations in the TCGA‐LIHC cohort.

### 2.14 Analysis of HCC subtypes based on prognostic biomarker expression

We conducted unsupervised clustering analysis of HCC samples using the consensusclusterplus r package (v1.64.0). Finally, we investigated the differences in OS and disease‐free survival rates between different groups using Kaplan–Meier curves.

### 2.15 Nomogram construction and prediction of drug sensitivity

A nomogram model was constructed using the rms r package (v6.7.1) to predict 1‐, 3‐, and 5‐year survival rates for patients with HCC. Calibration curves were employed to validate the accuracy of the nomogram in predicting patient prognosis. Furthermore, the oncopredict r package (v0.2) [38] was employed to evaluate the median inhibitory concentration (IC50) of various chemotherapy drugs in both high‐ and low‐score groups. The Wilcoxon rank‐sum test was utilized to analyze differences in the median IC50 values between high‐ and low‐score groups.

### 2.16 Real‐time quantitative PCR and proteomics analysis

Total RNA was extracted using TRIzol™ Reagent (15 596 018; Invitrogen, Carlsbad, CA, USA) and converted into cDNA using the ReverTra Ace qPCR RT master mix with a gDNA Remover kit (FSQ‐201; Toyobo, Osaka, Japan), followed by performing real‐time qPCR using Hieff® qPCR SYBR Green Master Mix (11201ES08; Yeasen, Shanghai, China). Experimental procedures followed the methods described by Shi et al. [39], and the primer sequences for qPCR are listed in Table S3. The protein levels of prognostic genes were analyzed using the cProSite database (https://cprosite.ccr.cancer.gov/).

### 2.17 H&E staining

Mouse liver tissues were fixed in 4% formalin at 4 °C overnight, followed by paraffin embedding and sectioning into 5 μm‐thick slices. The sections were dried in an oven at 45 °C for 1 h, then immersed in xylene I and xylene II for 10 min each to deparaffinize. Subsequently, they were rehydrated by immersing in anhydrous ethanol I and II for 1 min each, followed by 95% ethanol I and II for 1 min each. The sections were then immersed in 75% ethanol for 1 min and rinsed with distilled water for 1 min. Next, the sections were stained with hematoxylin solution (BSBA‐4021A; ZSGB‐BIO, Beijing, China) for 30 s to 1 min, followed by rinsing with distilled water for 15 min. The sections were then stained with eosin solution (ZLI‐9613; ZSGB‐BIO, Beijing, China) for 1 min. The sections were dehydrated by immersing in 95% ethanol III and IV for 1 min each, followed by immersion in anhydrous ethanol III and IV for 1 min each. Finally, the sections were immersed in xylene III and IV for 1 min each and sealed with neutral resin.

### 2.18 Immunohistochemistry

For the IHC assay, we used the ready‐to‐use fast IHC DAB Detection Kit (Polymer) (Kit‐0014; MXB Biotechnologies, Fuzhou, China). The sections were first dewaxed, dehydrated, and subjected to antigen retrieval in a microwave for 15 min using EDTA antigen retrieval solution (pH = 9.0) (ZLI‐9079; ZSGB‐BIO, Beijing, China). Then, the sections were incubated with primary antibodies against CD74 (Rabbit, 1 : 200, A24027; ABclonal, Wuhan, China) and CXCR4 (Mouse, 1 : 600, 60042‐1‐Ig; Proteintech, Wuhan, China) at 4 °C overnight. The blocking of endogenous peroxidases and DAB staining were performed using the DAB Detection Kit. Hematoxylin was used for counterstaining.

### 2.19 Statistical analysis

Statistical analyses were performed using both parametric and nonparametric approaches based on data distribution characteristics. For normally distributed data with independent samples, the unpaired two‐tailed Student's *t*‐test was applied, while the paired two‐tailed Student's *t*‐test was used for normally distributed paired samples. For data without a normal distribution, the unpaired two‐tailed Wilcoxon rank‐sum test was used for independent samples, whereas the Wilcoxon matched pairs signed rank test was applied for paired samples. Pearson's correlation coefficients were used to assess the correlations between two continuous variables. The log‐rank test was used to examine the difference in survival curves between the two groups. The differences in the proportions of cell subtypes between the high‐ and low‐risk groups were analyzed using the chi‐squared test. In this study, all statistical analyses were performed using r (v4.3.1) and graphpad prism (v9.5.0; GraphPad Software, San Diego, CA, USA) and statistical significance was set at *P* < 0.05.

## 3 Results

### 3.1 Identification at the single‐cell level in primary and early‐relapse HCC

**Fig. 1:**
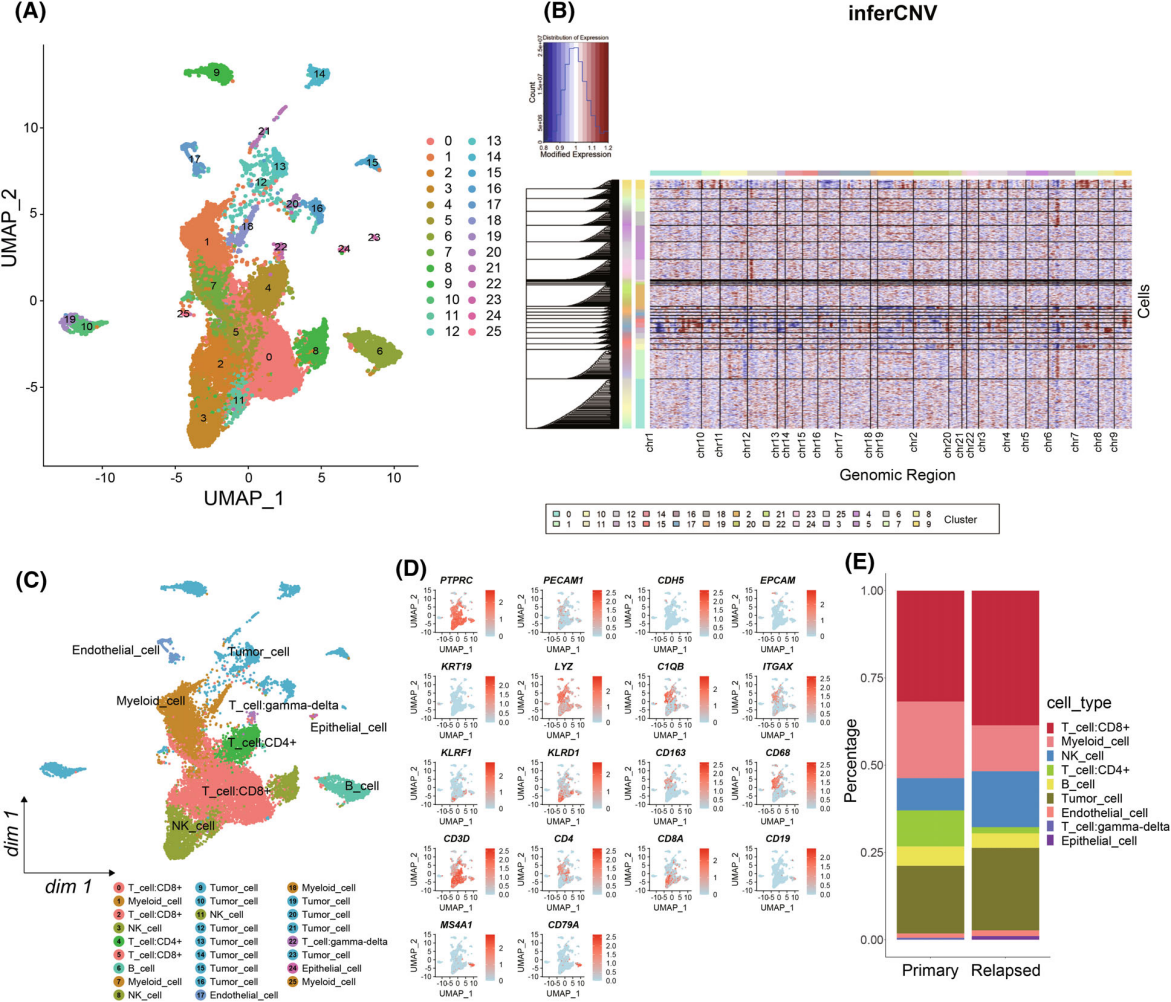
ScRNA‐seq identification of the primary and relapsed HCC. (A) UMAP plot of all cell clusters. (B) The chromosomal landscape of large‐scale CNVs inferred in individual cells enables us to distinguish malignant cells from nonmalignant ones (Malignant cells: Clusters 9, 10, 12, 13, 14, 15, 16, 19, 20, 21, 23). (C) UMAP plot of major cell types further identified by canonical markers. (D) UMAP plot showing the expression of marker genes for major cell types. (E) Histogram indicating the proportion of cells in primary and relapsed tumor samples. CNVs, copy number variations; HCC, hepatocellular carcinoma; scRNA‐seq, single‐cell RNA sequencing; UMAP, uniform manifold approximation and projection.

This study conducted a comprehensive analysis using public single‐cell sequencing results from 12 primary and 6 early‐relapse patients with HCC [5]. A total of 16 498 cells were obtained after strict data quality control and filtering. We employed Seurat to perform clustering analysis on these cells, resulting in a total of 25 cellular subgroups, and we further characterized the spatial landscape of all subgroups of HCC by dimensional reduction using a manifold learning method of UMAP (Fig. 1A). Figure S1A illustrated the distribution of samples from primary and relapsed HCC. We initially annotated the subgroups using SingleR (Fig. S1B). Additionally, the majority of nonimmune cell subgroups identified by SingleR were defined as malignant cells (tumor cells) by inferCNV (Fig. 1B, Fig. S1C). We further annotated and identified the major cell subclusters using marker genes and visualized them using UMAP and TSNE (Fig. 1C, Fig. S1D). All immune cells were PTPRC^+^ (Fig. 1D, Fig. S1E). The identified immune cells primarily consisted of myeloid cells (LYZ and C1QB), T cells (CD3D, CD3G, CD4, and CD8A), B cells (MS4A1 and CD79A), and natural killer (NK) cells (KLRF1 and KLRD1). Meanwhile, nonimmune cells mainly included endothelial cells (ECs: PECAM1 and CDH5), apparently normal epithelial cells (EPCAM and KRT19), as well as HCC malignant cells, etc. (Fig. 1C,D, Fig. S1E). Furthermore, we found that in early‐relapse HCC, there was an increase in the proportions of CD8^+^ T cells, NK cells, and malignant tumor cells, while the proportions of CD4^+^ T cells and myeloid immune cells decreased (Fig. 1E). This indicated heterogeneity in the ecosystem between early‐relapse and primary HCC.

### 3.2 Cell–cell interaction analysis related to tumor cells

**Fig. 2:**
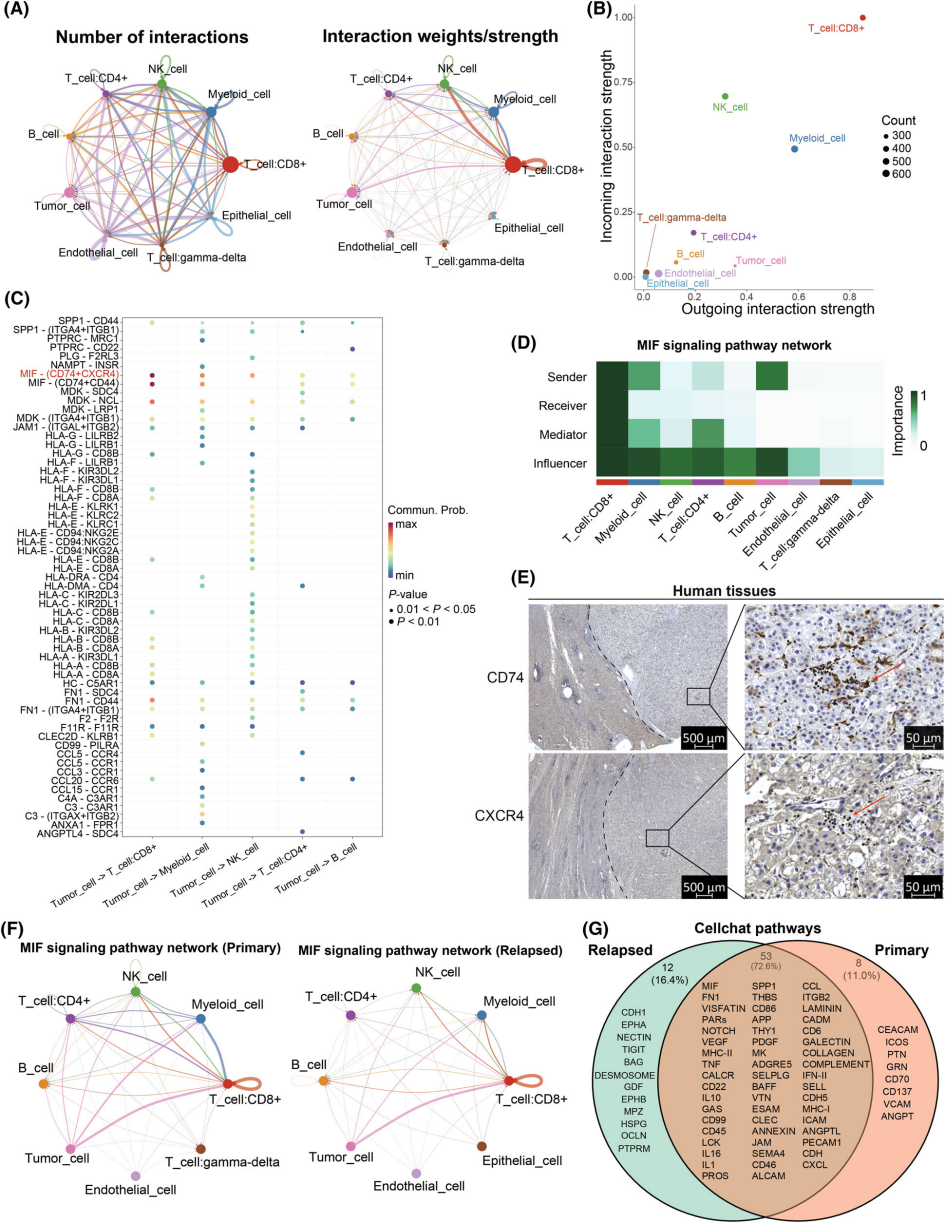
Analysis of cell–cell interactions. (A) The number and weights/strength of interactions in the cell–cell communication network. (B) Scatter diagram showing the incoming and outgoing pathway strength of the cell clusters. (C) Bubble plot showing interactions between tumor cells and major immune cells mediated by multiple ligand‐receptor pairs or signaling pathways. (D) The heatmap showing the relative importance of each cell cluster based on the computed network centrality measures. (E) CD74 and CXCR4 expression in human HCC and adjacent normal tissues by IHC experiments. The left side of the dashed line shows the adjacent normal tissues, while the right side shows the HCC tumor tissues. Representative IHC staining images from samples of 12 patients with HCC at 20× magnification; 500 or 50 μm scale bar. (F) Network diagram of intercellular communication mediated by the MIF signaling pathway in PT and RT tissues. (G) The Venn diagram illustrating the shared and distinct ligand‐receptor signaling pathways in RT and PT tissues. HCC, hepatocellular carcinoma; IHC, immunohistochemistry; MIF, macrophage migration inhibitory factor; PT, primary tumor; RT, relapsed tumor.

The occurrence and development of tumors constitute a complex dynamic procedure, continually characterized by interactions between the tumor cells and matrix/immune cells in the tumor microenvironment [40]. We employed CellChat [27, 28] to investigate ligand‐receptor interactions among malignant cells and other cells in the tumor microenvironment (see Section 2). The cellular interaction network diagram demonstrated the number and weight of interactions between various types of cells, with the strongest interactions between tumor cells and several major immune cells, including myeloid cells, CD8^+^ T cells, CD4^+^ T cells, NK cells, and B cells (Fig. 2A, Fig. S2A). Meanwhile, these types of immune cells and tumor cells were also the primary participants as both senders and receivers of cellular interaction signals (Fig. 2B). The ligand‐receptor interactions between tumor cells and major immune cell populations were mainly present in the MIF signaling pathway, with the ligand‐receptor pair MIF‐(CD74 + CXCR4) exerting the most significant influence on this pathway (Fig. 2C and Fig. S2B). This suggested that tumor cells primarily affected the functions of most immune cells through the MIF signaling pathway, thereby influencing the occurrence of tumors and the survival status of patients with liver cancer. Interestingly, the clinical data analysis from TCGA further validated these findings, revealing that patients with HCC with high expression of the *CXCR4* or *MIF* genes exhibit a trend toward shorter OS time and poorer prognosis (Fig. S2C,D). Tumor cells primarily played the roles of signal senders and influencers in the MIF signaling pathway (Fig. 2D), suggesting that in HCC, tumor cells can make a direct influence on immune cells. Consequently, this led the majority of immune cells to assume a complex dual role, thereby exhibiting both antitumor and pro‐tumor effects. IHC analysis revealed that, compared with adjacent normal tissues, the overall protein level of CD74 was decreased in HCC tumor tissues, while CXCR4 showed no significant difference. However, both CD74 and CXCR4 were highly expressed in the infiltrating immune cells within the tumor tissues (Fig. 2E). Moreover, we further analyzed the mRNA levels of *Cd74* and *Cxcr4* in tumor tissues of the mouse Hepa1‐6 implantation models (Fig. S2E,F). Furthermore, cellular interaction analyses conducted separately on early‐relapse and primary tumors revealed that the most significant signaling pathway mediating interactions between tumor cells and the main immune cell populations in the tumor microenvironment of both types of HCC was the MIF signaling pathway (Fig. S2G,H). However, there was a certain difference in the strength of the MIF signaling pathway among cell subpopulations between the two types of HCC tumors (Fig. 2F). Finally, we also discovered that there were differences in the number and types of cellular interaction signaling pathways within the microenvironments of early‐relapse and primary tumors (Fig. 2G), where unique ligand‐receptor signaling pathways in early‐relapse tumors (including TIGIT and HSPG) might play a key role in the recurrence process of HCC.

### 3.3 Tumor cells demonstrate multifaceted heterogeneity in relapsed and primary HCC

**Fig. 3:**
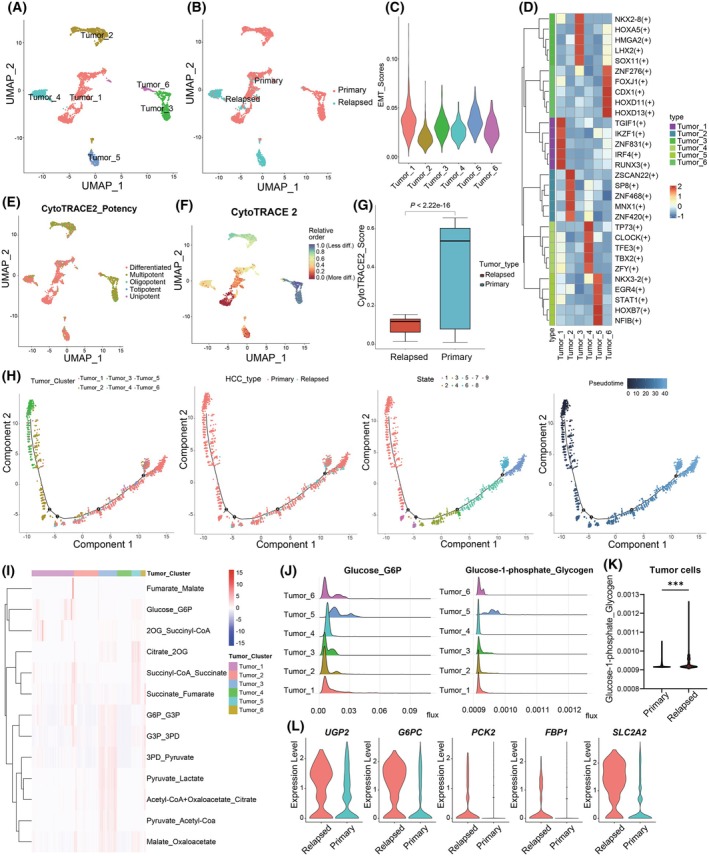
Analysis of tumor cell heterogeneity in relapsed and primary HCC. (A) UMAP plot of subclustered tumor cells, labeled in different colors. (B) UMAP plot of relapsed and primary tumor cells separated by disease state. (C) Violin plot indicating the EMT scores of tumor cell subgroups. (D) Heatmap showing the AUC scores of transcription factor motifs estimated per tumor cell by SCENIC. The top five differentially activated motifs in each tumor cell subpopulation are shown. (E, F) UMAP embedding of CytoTRACE 2 potency scores, indicating the absolute developmental potential of each tumor cell. (G) The boxplot displaying the CytoTRACE 2 potency scores of tumor cells in PT and RT (*n* = 2046 tumor cells from 12 primary tumor samples; *n* = 872 tumor cells from six relapsed tumor samples). The *P* value was calculated by the unpaired two‐tailed Wilcoxon rank‐sum test. The box represents the IQR, with the central line indicating the median expression level. The whiskers extend to the minimum and maximum values within 1.5 times the IQR from the first and third quartiles, respectively. (H) Pseudotime‐ordered analysis of Tumor cells from PT and RT tumor samples. (I) Heatmap of the predicted fluxome of 13 glycolytic and TCA cycle modules. Here, each row represents the flux between two metabolites for all the tumor cells of the six subgroups. (J) Distribution of predicted fluxome of glycogen synthesis modules in each tumor cell subgroup. (K) Violin plot displaying the predicted fluxome of glycogen synthesis modules in tumor cells from PT and RT samples, with median and interquartile ranges (*n* = 2046 tumor cells from 12 primary tumor samples; *n* = 872 tumor cells from six relapsed tumor samples). The *P* value was calculated by the unpaired two‐tailed Wilcoxon rank‐sum test. (L) Violin plot illustrating the expression of genes related to glycogen synthesis and gluconeogenesis in tumor cells from RT and PT samples. Statistical difference was denoted as ***P < 0.001. AUC, area under the curve; EMT, epithelial–mesenchymal transition; HCC, hepatocellular carcinoma; IQR, interquartile range; PT, primary tumor; RT, relapsed tumor; TCA, tricarboxylic acid; UMAP, uniform manifold approximation and projection.

Considering the important role of tumor cells in the tumor microenvironment, we next focused on tumor cells (total of 2918 cells) by extracting only the population of cells with highly altered copy number. We pooled and reanalyzed this subset using Seurat, identifying a total of six tumor cell subgroups (Fig. 3A). Relapsed tumor cells primarily clustered in Subgroups 4 and 5, while primary tumor cells clustered mainly in Subgroups 1, 2, 3, and 6 (Fig. 3B). Therefore, this indicated that, compared with primary HCC, malignant cells in early‐relapse HCC exhibited significant differences and specificity in clustering. In general, by comparing EMT gene signatures in these tumor cell subgroups, it was found that Subgroups 1 and 5 had the highest EMT scores, while Subgroup 2 had the lowest (Fig. 3C). Through SCENIC analysis, a computational method to infer gene regulatory networks and cell types from single‐cell RNA‐seq data, we confirmed that these six tumor cell subgroups each exhibited specific key transcription factors that may regulate their development (Fig. 3D and Fig. S3A). This further validated the accuracy of our tumor cell clustering. The transcription factors TBX2 and NFIB were highly activated in the relapsed tumor subgroups (Subgroups 4 and 5), potentially playing profound roles in transcriptional regulation in the recurrence of HCC tumors (Fig. 3D).

To further investigate the differentiation heterogeneity between relapsed and primary tumor cells, we analyzed the absolute developmental potential of tumor cell subgroups using CytoTRACE and combined this with Monocle to infer the dynamic states and transition trajectories of tumor cells (see Section 2). We found that tumor cell Subgroups 2 and 3 exhibited the highest differentiation potential, a phenotype that may be linked to their increased proliferation and invasion capabilities (Fig. 3E,F). Overall, primary tumor cells exhibited higher CytoTRACE scores than relapsed tumor cells and relapsed tumor cells are mainly at the end of the trajectory path (Fig. 3G,H), raising the question of whether relapsed tumor cells may partially originate from residual primary tumor cells. Ding et al. revealed that 52% of relapsed HCCs derive from a clonal lineage of the initial tumor through comparison of genetic characteristics [41]. Previous studies reported that recurrence in the remnant liver after resection typically occurs due to intrahepatic metastasis originating from the primary tumor or due to a multicentric genesis [42, 43, 44].

It is well known that metabolic abnormalities are one of the key characteristics of tumors. To assess the metabolic heterogeneity of relapsed and primary tumor cells, we evaluated the metabolic flux of all tumor cells using scFEA (see Section 2). We observed the flux levels of 13 glycolysis and TCA cycle modules in all tumor cell subpopulations (Fig. 3I, Fig. S3B,C). Subgroup 3 exhibited the highest levels of metabolic activity in glycolysis and the TCA cycle (Fig. 3I). A previous study has demonstrated that abnormalities in glycogen metabolism can drive the initiation and progression of liver cancer [45]. We further explored the glycogen synthesis flux in relapsed and primary tumor cells, discovering that Subpopulation 5, primarily composed of relapsed tumor cells, exhibited the highest level of glycogen synthesis flux (Fig. 3J). Additionally, the glycogen synthesis flux in relapsed tumor cells was generally higher than that in primary tumor cells (Fig. 3K). We further analyzed the expression levels of genes associated with glycogen synthesis and gluconeogenesis between primary and relapsed tumor cells. Interestingly, the expression levels of *UGP2*, *G6PC*, *PCK2*, *FBP1*, and *SLC2A2* were significantly higher in relapsed tumors (Fig. 3L). The above findings suggest that abnormalities in glycogen metabolism may be a key factor in the recurrence of HCC.

### 3.4 Transcriptomic heterogeneity and potential therapeutic targets in relapsed HCC

**Fig. 4:**
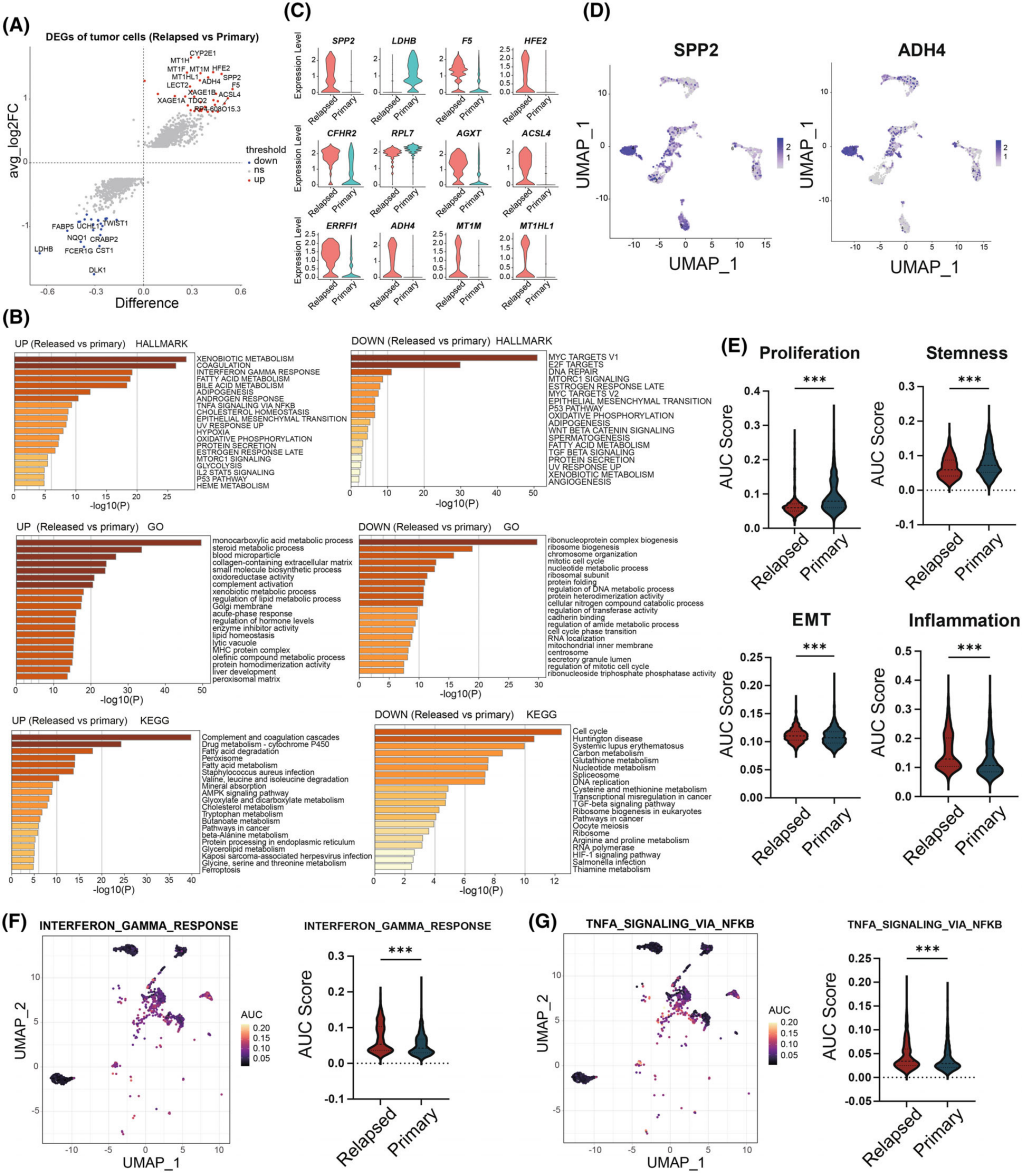
Tumor cell transcriptomic heterogeneity between primary and relapsed HCC. (A) Volcano plot showing differentially expressed genes between relapsed and primary malignant cells. The names of the most significant genes are indicated in the plots. (B) Bar chart showing the enrichment of specific pathways in primary or recurrent malignant cells, including enrichment analysis based on Hallmark Gene Sets, GO enrichment analysis, and KEGG enrichment analysis. (C) Violin plot showing the expression of top 12 DEGs between primary and relapsed malignant cells in all cell types. (D) UMAP plot demonstrating SPP2 and ADH4 expression in relapsed and primary malignant cells. (E) Violin plots illustrating individual tumor cell AUC score overlay for proliferation, stemness, EMT, and inflammation in relapsed HCC (*n* = 872 tumor cells from six relapsed tumor samples) vs. primary HCC (*n* = 2046 tumor cells from 12 primary tumor samples), with median and interquartile ranges. The *P* values were calculated by the unpaired two‐tailed Wilcoxon rank‐sum test. (F, G) Individual tumor cell AUC score overlay for interferon gamma response and TNFα signaling via NFκB pathway activities, comparing relapsed HCC with primary HCC (*n* = 2046 tumor cells from 12 primary tumor samples; *n* = 872 tumor cells from six relapsed tumor samples). Statistical differences were calculated by the unpaired two‐tailed Wilcoxon rank‐sum test. Data are presented as violin plots with median and interquartile ranges. Statistical difference was denoted as ***P < 0.001. AUC, the area under the curve; DEGs, differentially expressed genes; EMT, epithelial–mesenchymal transition; GO, Gene Ontology; HCC, hepatocellular carcinoma; KEGG, Kyoto Encyclopedia of Genes and Genomes; UMAP, uniform manifold approximation and projection.

The above suggested that the heterogeneity of the tumor ecosystem between primary tumors (PT) and relapsed tumors (RT) might stem from differences in malignant cells. Therefore, it was crucial to further explore the transcriptomic differences between malignant cells in primary and relapsed tumors. To investigate these differences, we performed a DEG analysis between RT and PT malignant cells from the discovery cohort. Notably, the genes upregulated in RT malignant cells primarily included *SPP2*, *ADH4*, *HFE2*, *CYP2E1*, along with metallothionein (MT) genes, specifically *MT1F*, *MT1M*, and *MT1H* (Fig. 4A). Previous studies have indicated that MTs played a crucial role in the formation, metastasis, and drug resistance of tumors [46, 47, 48, 49, 50]. GO, KEGG enrichment analysis, and enrichment analysis based on the HALLMARK gene set demonstrated that genes upregulated in RT were primarily involved in immune response‐related pathways, such as INTERFERON GAMMA RESPONSE, TNFA SIGNALING VIA NFKB, and COMPLEMENT AND COAGULATION CASCADES (Fig. 4B). Whereas in PT, upregulated genes predominantly participated in cell cycle‐related pathways, including MYC TARGETS V1, E2F TARGETS, RIBONUCLEOPROTEIN COMPLEX BIOGENESIS, and CELL CYCLE (Fig. 4B). Furthermore, it is noteworthy that among the top 12 protein‐coding genes showing the most significant differences in malignant cells between PT and RT, several genes, notably including *SPP2* and *ADH4*, were significantly upregulated in relapsed malignant cells and exhibited higher expression in malignant cells compared with other cell types (Fig. 4C,D, Fig. S4A,B). Figure S4C illustrates the marker genes of malignant cells. Interestingly, we observed that the expression levels of *SPP2* and *ADH4* were significantly lower in tumor samples compared with normal samples in the TCGA database (Fig. S4D). This difference was consistent with the protein levels observed in the cProSite database (Fig. S4E). To further validate the mRNA expression levels of these two genes in HCC, we established the mouse orthotopic implantation models. The representative images of H&E staining confirmed the successful establishment of the models (Fig. S4F). RT‐qPCR experiments demonstrated that in mouse liver cancer tissues, the expression levels of *Spp2* and *Adh4* were significantly lower in tumor tissues compared with adjacent nontumor tissues (Fig. S4G). This discrepancy in results further highlights the advantages of single‐cell sequencing in identifying cellular heterogeneity. Furthermore, we observed that patients with HCC with higher expression levels of *SPP2* and *ADH4* exhibited significantly better survival outcomes (Fig. S4H,I). These findings suggested that *SPP2* and *ADH4* might have played distinct roles in the progression and metastasis of malignant cells in relapsed liver cancer, thereby holding promise as therapeutic targets for relapsed HCC. We investigated the proliferation, stemness, EMT, and inflammation levels in malignant cells between PT and RT HCC by area‐under‐the‐receiving operating characteristic analysis (see Section 2). We discovered that primary malignant cells exhibited significantly higher levels of proliferation and stemness activity compared with relapsed malignant cells, whereas their inflammation and EMT levels were markedly lower than those observed in the relapsed malignant cells (Fig. 4E). This was consistent with the results of the previous CytoTRACE analysis. This suggests that primary malignant cells possess a greater capacity for growth and self‐renewal and provoke a lesser inflammatory response in the surrounding tissue environment than their relapsed counterparts. In our further exploration of inflammation‐related pathway activities in malignant cells between primary and relapsed HCC, we observed that the activities of interferon gamma response and TNFα signaling via NFκB pathways were significantly higher in malignant cells from RT compared with PT (Fig. 4F,G). The above indicated that malignant cells from RT were in a higher inflammatory and differentiation state.

### 3.5 Machine learning‐based screening of risk predictors for RTRS

**Fig. 5:**
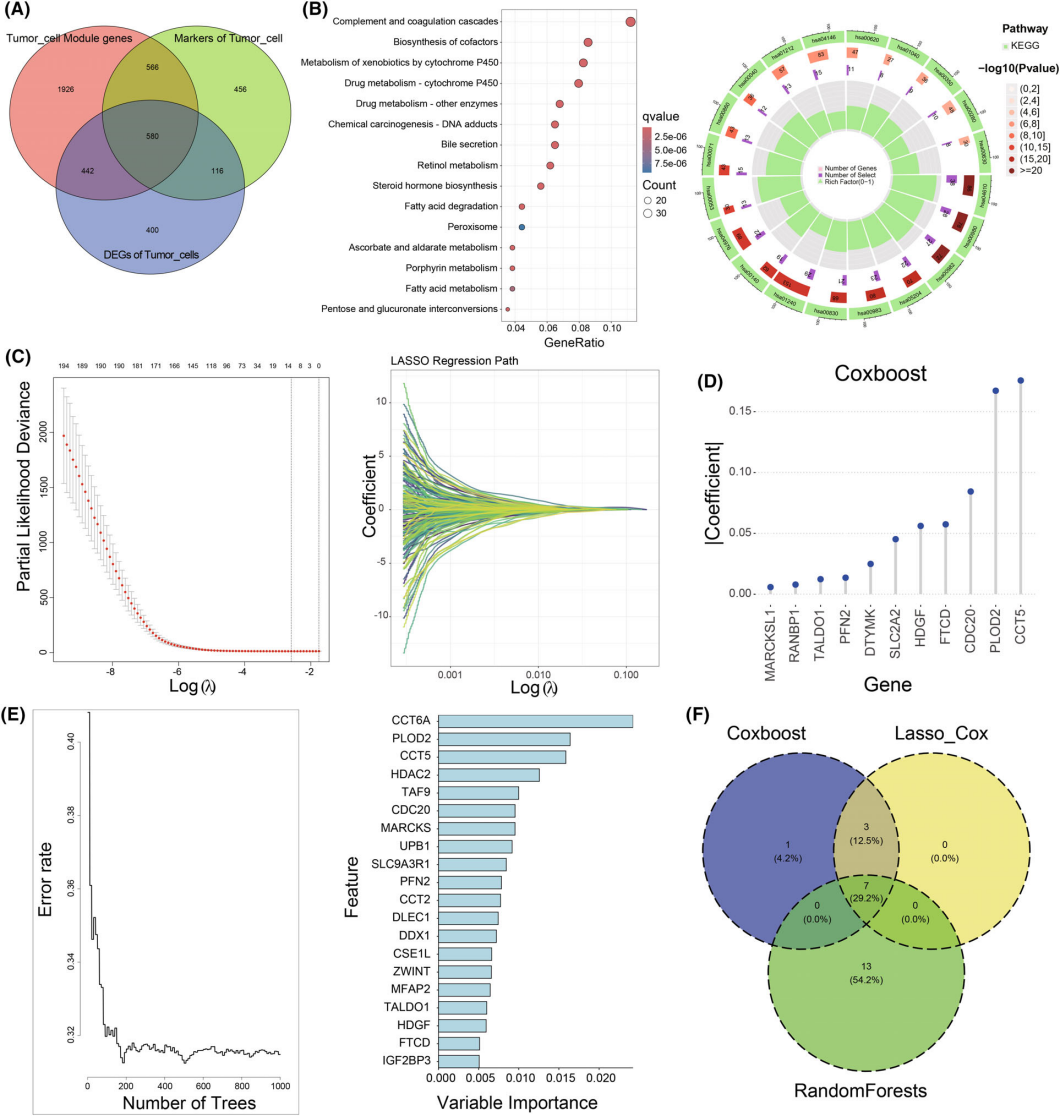
Machine learning‐based screening of key prognostic factors. (A) Venn diagram illustrating the overlap among differentially expressed genes in malignant cells between RT and PT, malignant cell marker genes identified through scRNA‐seq analysis, and malignant cell‐related module genes obtained from hdWGCNA. (B) Bubble plot and circle plot of GO enrichment analysis for the overlap genes. (C) Dimension reduction of the 195 potential prognostic genes by the Lasso algorithm. (D) Dimension reduction of the 195 potential prognostic genes by the CoxBoost algorithm. (E) Dimension reduction of the 195 potential prognostic genes by the Random Survival Forest algorithm. (F) The Venn diagram illustrating the intersection of prognostic genes obtained by the three machine learning methods, respectively. GO, Gene Ontology; PT, primary tumor; RT, relapsed tumor; scRNA‐seq, single‐cell RNA sequencing.

To identify prognostic relevant genes for relapsed tumor cell‐related risk score (RTRS), we initially employed hdWGCNA to perform gene co‐expression network analysis on HCC single‐cell sequencing data using an optimal soft thresholding power of 10 (Fig. S5A,B). In total, we identified a total of 20 unique gene co‐expression modules, among which we discovered that modules, such as M1, M2, M6, M7, M9, M10, M18, M19, and M20, were significantly correlated with tumor cells, whereas the M4 module had a highly significant correlation with immune cells (Fig. S5C,D). Subsequently, we intersected DEGs in tumor cells between early relapse and primary HCC, marker genes of tumor cells, and genes from modules significantly associated with tumor cells identified through hdWGCNA analysis (Fig. 5A). KEGG and GO enrichment analyses of the 580 intersected genes demonstrated that these genes predominantly participate in signaling pathways, including complement and coagulation cascades and the biosynthesis of cofactors (Fig. 5B, Fig. S6A,B). Subsequently, 195 potential prognostic genes were identified by univariate Cox regression analysis (Fig. S6C). Three machine learning algorithms for survival, including LassoCox (Fig. 5C), CoxBoost (Fig. 5D), and RSF (Fig. 5E), were further applied to determine seven intersecting prognostic genes (*PFN2*, *PLOD2*, *CDC20*, *CCT5*, *TALDO1*, *HDGF*, and *FTCD*) (Fig. 5F).

### 3.6 Construction of the RTRS model

**Fig. 6:**
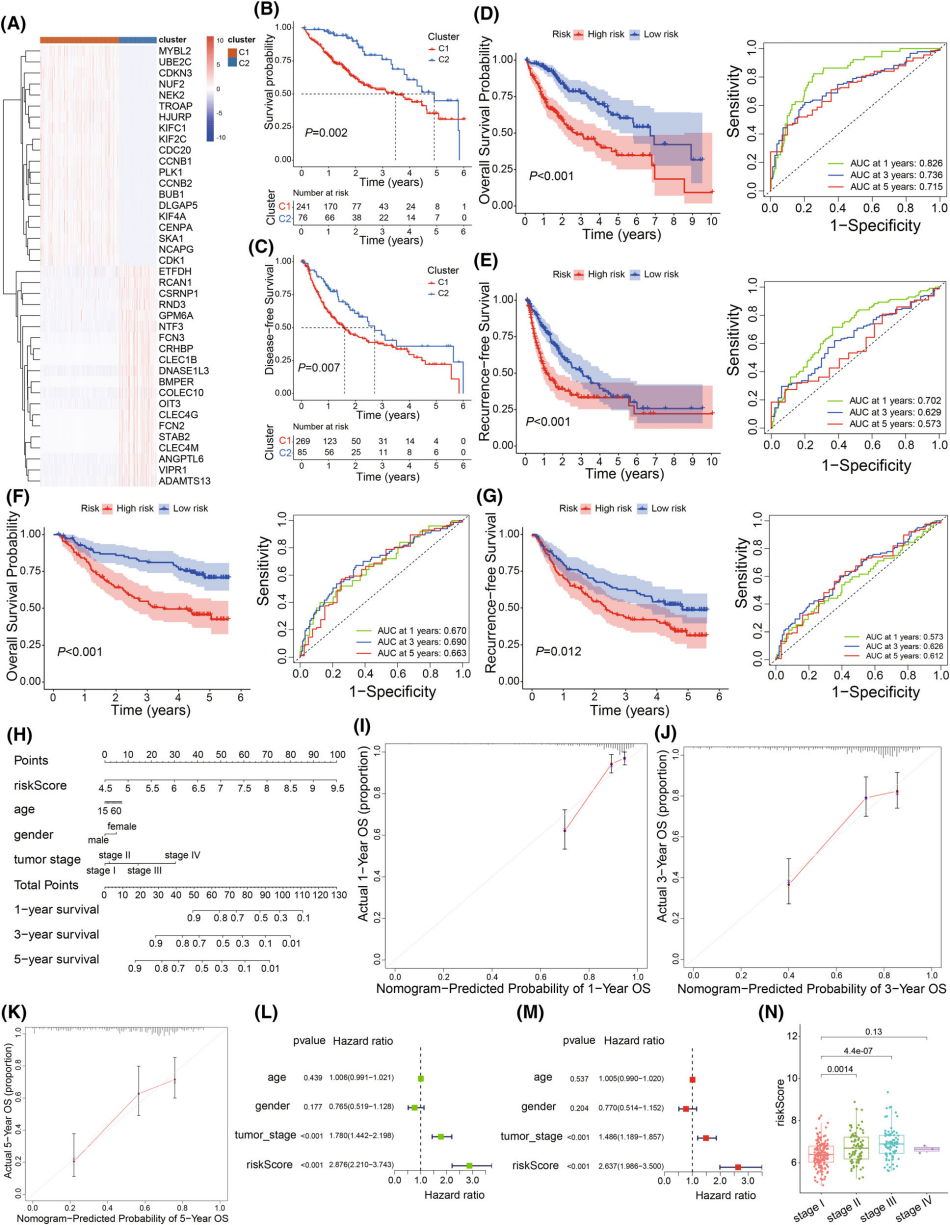
Development and validation of the RTRS. (A) Heatmap of the top 40 differentially expressed genes between two clusters (*n* = 284 samples for cluster C1; *n* = 87 samples for cluster C2). The clustering was based on unsupervised consensus clustering analysis. (B, C) Survival curve of OS and RFS between two clusters (*n* = 284 samples for Cluster C1; *n* = 87 samples for Cluster C2). Log‐rank *P* values are indicated. (D, E) Kaplan–Meier curve of OS or RFS for two groups in the TCGA‐LIHC training cohort (*n* = 171 samples for the high‐risk group and *n* = 172 samples for the low‐risk group). Log‐rank *P* values are indicated. The shaded areas around each curve indicate the 95% confidence intervals for the survival estimates. Time‐dependent ROC curves of 1‐, 3‐, and 5‐year OS or RFS. (F, G) Kaplan–Meier curve of OS or RFS for two groups in the GSE14520 validation cohort (*n* = 110 samples for high‐risk group; *n* = 111 samples for the low‐risk group). Log‐rank *P* values are indicated. The shaded areas around each curve indicate the 95% confidence intervals for the survival estimates. Time‐dependent ROC curves of 1‐, 3‐, and 5‐year OS or RFS. (H) Nomogram was established in the TCGA‐LIHC cohort with riskScore and clinical factors (*n* = 318 patients). (I–K) The calibration plots of the nomogram illustrate the concordance between predicted and observed survival rates at 1, 3, and 5 years (*n* = 318 patients). The red line indicates perfect prediction, with error bars indicating the 95% confidence intervals. (L) Univariate and (M) multivariate cox regression analysis (*n* = 318 patients). The hazard ratio is presented as a point estimate, and the error bars represent the 95% confidence interval. (N) The boxplot shows the riskScore levels across tumor stages in the TCGA‐LIHC cohort (*n* = 159 samples for stage I; *n* = 76 samples for stage II; *n* = 80 samples for stage III; *n* = 3 samples for stage IV). The box represents the IQR, with the central line indicating the median expression level. The whiskers extend to the minimum and maximum values within 1.5 times the IQR from the first and third quartiles, respectively. The *P* values were measured using the unpaired two‐tailed Wilcoxon rank‐sum test. IQR, interquartile range; OS, overall survival; RFS, recurrence‐free survival; RTRS, relapsed tumor cell‐related risk score.

Firstly, to validate the reliability and stability of these recurrence‐related prognostic factors, we performed an unsupervised consensus clustering analysis to accurately classify the TCGA‐LIHC cohort into two distinct subtypes (Fig. S7A–C). Figure 6A presented the top 40 DEGs between the two clusters. Additionally, it is noteworthy that Kaplan–Meier survival analyses of both OS and RFS revealed that Cluster C1 had a worse prognosis compared with Cluster C2, with *P* values of 0.002 and 0.007, respectively (Fig. 6B,C). The above results demonstrated that the seven postoperative recurrence‐related prognostic factors had a significant stratification effect on patients with HCC. Next, to further explore the prognostic value of these risk factors, we established a RTRS for predicting RFS time and categorized patients into high and low‐score groups. In both the training (TCGA) and validation (GSE14520) cohorts, the high‐score group exhibited a worse prognosis for OS and RFS (Fig. 6D–G). Moreover, ROC curve analysis revealed that these seven prognostic genes are high‐quality risk predictors for both OS and RFS patients with HCC (Fig. 6D–G).

To explore the potential value and predictive effect of the clinical application of the RTRS model, we analyzed the risk scores in conjunction with other clinical characteristics and constructed a new nomogram model (Fig. 6H). The C‐index of the nomogram model is 0.73, and the calibration curve demonstrated excellent concordance between the predicted and actual survival probabilities at 1‐, 3‐, and 5‐year intervals (Fig. 6I–K). Univariate and multivariate Cox models showed that the risk score was an independent prognostic predictor (Multivariate Cox model: HR = 2.637, *P* < 0.001), and the risk score was more effective in predicting prognosis than individual clinical characteristics (Fig. 6L,M). There were significant differences in risk scores among patients with HCCwith different tumor stages (Fig. 6N). The trend indicates that higher tumor stages are associated with increased RTRS scores, suggesting that the RTRS offers robust diagnostic value in assessing tumor progression. Notably, while the association holds true for earlier stages, the limited number of stage IV samples precluded a definitive evaluation of the RTRS performance at this advanced stage. The above results further confirm the reliability and clinical applicability of the predictive model we constructed.

### 3.7 Immune microenvironment landscape and analysis of drug sensitivity

**Fig. 7:**
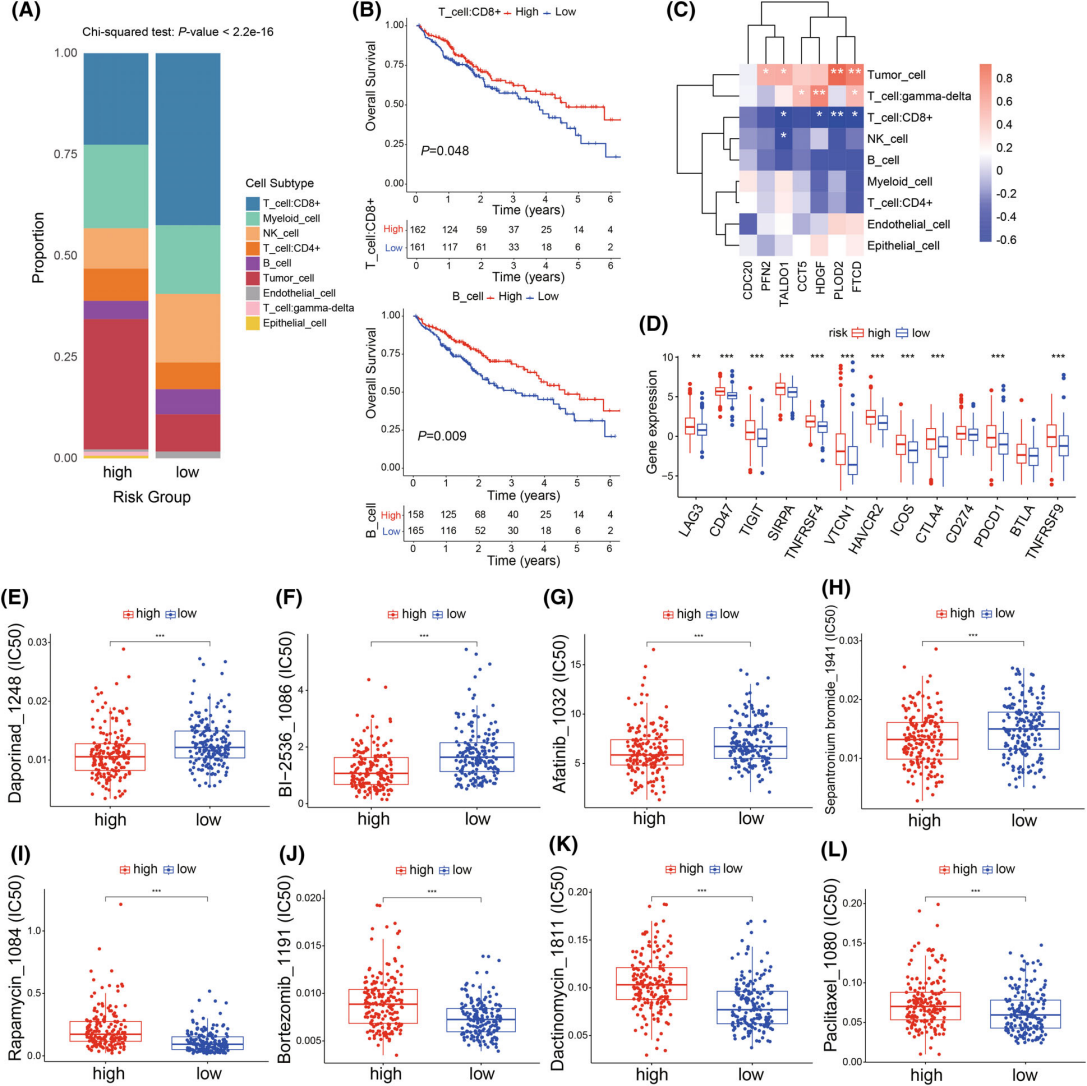
Analysis of immune microenvironment and drug sensitivity. (A) Histogram indicating the proportion of cells in the high‐score and low‐score groups from single‐cell RNA sequencing data. The *P* value was calculated using the Chi‐squared test. (B) Kaplan–Meier analysis demonstrating the impact of patient immune cell infiltration levels on overall survival rate (*n* = 171 samples for the high‐risk group and *n* = 172 samples for the low‐risk group). The cell proportions were deconvoluted from TCGA‐LIHC cohort using HCC single‐cell data for reference. Significance was calculated by the log‐rank test. (C) Heatmap showing the correlation between prognostic genes and cell subtypes. The Pearson's correlation coefficient was employed to calculate the correlation. (D) Boxplots illustrating the expression levels of immune checkpoints in the high‐score (*n* = 171 samples) and low‐score (*n* = 172 samples) groups. The box represents the IQR, with the central line indicating the median expression level. The whiskers extend to the minimum and maximum values within 1.5 times the IQR from the first and third quartiles, respectively. Statistical differences were calculated by the unpaired two‐tailed Wilcoxon rank‐sum test. (E–L) Differences in chemotherapy drug sensitivity between two risk groups (*n* = 171 samples for the high‐risk group and *n* = 172 samples for the low‐risk group). The box represents the IQR, with the central line indicating the median expression level. The whiskers extend to the minimum and maximum values within 1.5 times the IQR from the first and third quartiles, respectively. The *P* values were measured using the unpaired two‐tailed Wilcoxon rank‐sum test. Statistical differences are denoted as **P < 0.01, ***P < 0.001. IQR, interquartile range.

To thoroughly investigate immune cell infiltration in high‐ and low‐score samples, we categorized patient samples from the scRNA‐seq data into high‐ and low‐risk groups. We observed that the low‐score group had higher proportions of CD8^+^ T cells, NK cells, and B cells, whereas the high‐score group showed a higher proportion of tumor cells (Fig. 7A). We observed that higher infiltration levels of CD8^+^ T cells and B cells were significantly associated with better survival outcomes (Fig. 7B), aligning with the increased proportions of these cells in the low‐risk group. Additionally, we found significant negative correlations between these risk predictors and CD8^+^ T cells (Fig. 7C). Subsequently, our analysis of immune checkpoint expression levels in high‐ and low‐score groups indicated that the expression of *LAG3*, *CD47*, *TIGIT*, *SIRPA*, *TNFRSF4*, *VTCN1*, *HAVCR2*, *ICOS*, *CTLA4*, *PDCD1*, and *TNFRSF9* immune checkpoints was significantly elevated in the high‐score group (Fig. 7D), which may be associated with the poorer survival prognosis observed in this group. This suggests that immunotherapy targeting immune checkpoints may be a suitable treatment approach for high‐score group patients with HCC.

Subsequently, to precisely select appropriate chemotherapy agents for patients with HCC classified as high‐ or low‐risk, we conducted a drug sensitivity analysis using oncoPredict [38]. It was observed that high‐risk patients demonstrated greater sensitivity to medications like Daporinad and Afatinib (Fig. 7E–H and Fig. S8A–D). This indicated that these drugs could potentially offer enhanced effectiveness in the chemotherapy of high‐risk patients, and their administration following surgery might assist in postponing the recurrence of HCC. The low‐risk group showed higher sensitivity to drugs like Rapamycin and Bortezomib (Fig. 7I–L and Fig. S8E–H). The findings contribute to guiding targeted therapeutic strategies for high‐ and low‐risk patients with HCC.

### 3.8 Validation of risk genes in HCC

**Fig. 8:**
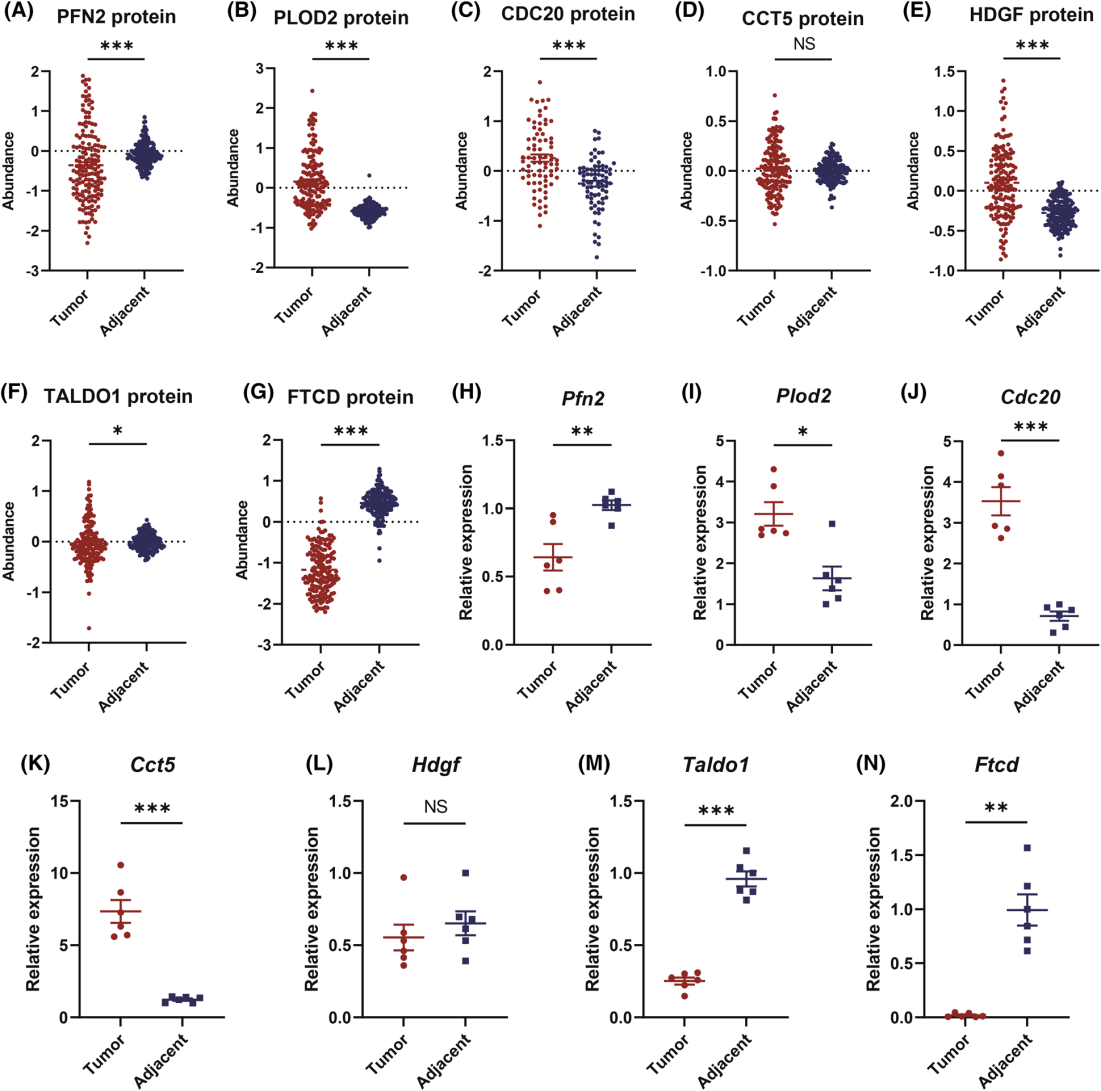
Proteomics and RT‐qPCR of the prognostic factors. (A–G) Protein levels of prognostic genes in HCC were assessed using the cProSite database. Data are shown as mean ± SEM (CDC20: *n* = 75 tumor/adjacent pairs from 75 patients with HCC; other proteins: *n* = 159 pairs from 159 patients). Statistical comparisons between tumor and adjacent tissues were performed using an unpaired two‐tailed Student's *t*‐test (D, E) or unpaired two‐tailed Wilcoxon rank‐sum test (A–C, F, G). (H–N) Differential mRNA expression of prognostic genes in tumor versus adjacent tissues from an orthotopic murine HCC model (*n* = 6 mice). Relative mRNA levels were quantified by RT‐qPCR with three technical replicates per sample. Data represent mean ± SEM (*n* = 6 biological replicates). Statistical significance between tumor and adjacent tissues were determined using paired two‐tailed Student's *t*‐test (H, J–M) or Wilcoxon matched pairs signed rank test (I, N). Statistical differences are denoted as *P < 0.05, **P < 0.01, ***P < 0.001, and NS for not significant. HCC, hepatocellular carcinoma; RT‐qPCR, real‐time quantitative reverse transcription PCR. SEM, standard error of the mean.

We analyzed the protein levels of the seven prognostic genes in patients with HCC using the cProSite database. We found that the protein levels of PLOD2, CDC20, and HDGF were significantly upregulated in the tumor samples of patients with HCC; the protein levels of PFN2, TALDO1, and FTCD were significantly downregulated in the tumor samples; whereas CCT5 showed no difference (Fig. 8A–G). Next, we examined the mRNA expression levels of these seven prognostic genes in tumor and adjacent nontumor tissues using the mouse Hepa1‐6 implantation models (Table S4). Our findings revealed that the mRNA levels of *Pfn2*, *Taldo1*, and *Ftcd* were significantly downregulated in tumor tissues of Hepa1‐6 implantation models. Conversely, *Plod2*, *Cdc20*, and *Cct5* genes were significantly upregulated in tumor tissues. For *Hdgf*, no significant variation was observed (Fig. 8H–N). This indicated that the protein and mRNA levels of these genes were not entirely consistent, potentially due to mRNA alternative splicing and posttranslational modifications of the proteins. Additionally, there also existed a certain degree of heterogeneity between the mouse Hepa1‐6 implantation models and patients with HCC, suggesting that the mouse Hepa1‐6 implantation models may only represent a specific subtype of patients with HCC.

## 4 Discussion

Although surgical resection remains the current optimal treatment for HCC, tumor recurrence persists as an ongoing challenge and a critical factor influencing postoperative outcomes [4, 51]. Compared with primary HCC tumors, relapsed HCC tumors exhibited significant temporal and spatial heterogeneity [5]. This heterogeneity can influence not only diagnosis but also tumor progression rates, immune cell infiltration levels, and responsiveness to therapeutic interventions. Furthermore, a large number of studies suggested intratumor heterogeneity (ITH), which was a prognostic marker in multiple cancers, had a significant impact on tumor aggressiveness and immunity, and patients with high ITH exhibited significantly poorer survival rates [52, 53, 54, 55, 56]. A comprehensive analysis of this heterogeneity was crucial for revealing the dynamic evolutionary process of relapsed tumors. In this study, we analyzed the ligand‐receptor interactions between HCC tumor cells and major immune cell subpopulations and explored the heterogeneity of these interactions between relapsed and primary HCC tumors. We discovered that the MIF signaling pathway, particularly the MIF‐(CD74 + CXCR4), was the most prevalent form of communication, suggesting that the MIF signaling pathway was likely to be a key way in which tumor cells affected immune cells, consequently leading to an immunosuppressive microenvironment. IHC analysis revealed that CD74 and CXCR4 were highly expressed in the infiltrating immune cells within the tumor tissues. In addition to tumor cells secreting MIF cytokines to regulate immune cells, other stromal cells in HCC also influence the immune microenvironment through the MIF signaling pathway [57]. Additionally, we discovered distinctive and significant signaling pathways, such as TIGIT and HSPG, in early‐relapse HCC. Several studies have demonstrated that TIGIT is an inhibitory receptor shared by T cells and NK cells, capable of suppressing the cytotoxic effects of NK and T cells against tumor cells, and it represents a novel target for immunotherapy [58, 59, 60, 61, 62]. The above suggests that these specific signaling pathways may be involved in the recurrence process of HCC.

The currently available algorithms for analyzing single‐cell data are capable of predicting the potential for cell differentiation and constructing developmental trajectories, which are particularly powerful when studying specific biological events across spatial and temporal dimensions [63, 64]. Here, we applied these to comprehensively explore the heterogeneity of tumor cells between relapsed and primary HCC. We found that primary tumor cells overall exhibit higher CytoTRACE scores than relapsed tumor cells, indicating greater developmental potential. We inferred the dynamic states and transition trajectories of tumor cells using Monocle and observed that relapsed tumor cells were predominantly located at the end of the trajectory path. This suggests the possibility of a differentiation relationship between relapsed and primary tumors. Previous studies reported that relapsed tumors most likely derived from subclinical metastases of the primary tumor [4, 41, 42, 43, 65]. Particularly, tumors occurring within 2 years of surgery were more likely to demonstrate the same clonal origins as the original tumor [66, 67]. Additionally, the heterogeneity in the differentiation status of tumor cells in RT and PT may indicate varying degrees of malignancy. In general, compared with primary tumor cells, relapsed tumor cells exhibited significantly higher levels of EMT and inflammation. It has been reported that EMT is involved in the early recurrence of HCC [68].

Tumorigenesis relies on the reprogramming of cellular metabolism [69, 70, 71, 72]. Therefore, the heterogeneity between relapsed and primary HCC may primarily stem from differences in the metabolic activity of their tumor cells. Interestingly, we discovered variations in the flux levels of 13 glycolysis and TCA cycle modules between primary and relapsed tumor cells. Furthermore, the glycogen synthesis flux was generally higher in relapsed tumor cells compared with primary tumor cells. Previous studies reported a significant association between abnormalities in glycogen metabolism and the progression of HCC [45, 73, 74, 75, 76]. However, the impact of glycogen on the recurrence of HCC has not yet been reported. This is a crucial aspect that we aim to explore comprehensively in our subsequent research.

With the development of high‐throughput sequencing technology and artificial intelligence, the joint analysis of multi‐omics data has become an effective method for analyzing disease heterogeneity, screening therapeutic targets, and prognostic biomarkers [77]. To identify biomarkers for preventing postoperative tumor recurrence and key targets to extend the RFS time of patients with HCC, seven biomarkers (*PFN2*, *PLOD2*, *CDC20*, *CCT5*, *TALDO1*, *HDGF*, and *FTCD*) associated with tumor postoperative recurrence were successfully screened using multiple machine learning analysis methods. Additionally, the RTRS models constructed on the basis of these key genes showed excellent predictive performance. The analysis of immune checkpoints (ICBs) revealed significantly higher expression levels of *LAG3*, *CD47*, *TIGIT*, *SIRPA*, *TNFRSF4*, *VTCN1*, *HAVCR2*, *ICOS*, *CTLA4*, *PDCD1*, and *TNFRSF9* in the high RTRS group compared with the low RTRS group. Therefore, immune function may be significantly suppressed in the high‐score group, leading to poorer survival prognosis for these patients. The varying efficacy of ICB therapy across patients and cancer types highlights the crucial need to identify those patients who might benefit from ICB, aiming to develop the most effective immunotherapy strategies [78].

This study has facilitated a deeper insight into the mechanisms associated with HCC recurrence and contributed to the development of effective therapeutic targets and prognostic models, providing a reference for achieving precision medicine in patients with HCC. However, this study still has some limitations; further exploration of the molecular mechanisms of therapeutic targets for relapsed HCC via molecular and cellular experiments is necessary.

## 5 Conclusion

In summary, this study comprehensively explored the heterogeneity of early‐relapse HCC tumors by single‐cell sequencing analyses and multiple machine learning methods. For the first time, we have clarified the heterogeneity of malignant cells between early‐relapse and primary HCC in aspects, such as intercellular communication, differentiation status, metabolic activity, and transcriptomics. Moreover, we developed a novel and effective RTRS model, which has been validated to have significant independent prognostic value. Our work contributes significant research value to precision medicine for relapsed patients with HCC.

## Conflict of interest

The authors declare no conflict of interest.

## Author contributions

W‐JW, S‐YL, DC, and H‐LP conceived and designed the study. W‐JW and FC performed the experiments. JW, FC, GC, and JZ collected the clinical samples. W‐JW, JW, RF, HW, WH, and T‐YT performed the data analysis. W‐JW, JW, XW, BL, YH, DC, and S‐YL interpreted the data. W‐JW drafted the manuscript. DC, H‐LP, S‐YL, and W‐JW revised and edited the manuscript. S‐YL, WH, and T‐YT coordinated the study. All authors have read and approved the final manuscript.

## Supporting information


**Fig. S1.** ScRNA‐seq analysis in primary and relapsed HCC samples, related to Fig. 1.
**Fig. S2.** Cell–cell communication analysis, related to Fig. 2.
**Fig. S3.** Characteristics of tumor cells in relapsed and primary HCC, related to Fig. 3.
**Fig. S4.** Heterogeneity of malignant cell transcriptome in RT and PT samples, related to Fig. 4.
**Fig. S5.** Identification of potential gene modules associated with tumor cells by hdWGCNA.
**Fig. S6.** Identification of key prognostic factors, related to Fig. 5.
**Fig. S7.** Unsupervised consensus clustering analysis, related to Fig. 6.
**Fig. S8.** Analysis of drug sensitivity, related to Fig. 7.

**Table S1.** Summary of Clinical Information for HCC Patients.

**Table S2.** The differentially expressed genes in malignant cells (Relapsed vs. Primary).

**Table S3.** Summary of RT‐qPCR Primer Information.

**Table S4.** Relative expression of prognostic genes measured by RT‐qPCR.

## Data Availability

The datasets analyzed in this study are publicly accessible from the following repositories. Single‐cell RNA sequencing data for HCC were obtained from the China National GeneBank DataBase (https://db.cngb.org, accession number: CNP0000650), bulk RNA‐seq and clinical data for the TCGA‐LIHC dataset were retrieved from TCGA (https://cancergenome.nih.gov/), and the transcriptomic dataset GSE14520 was downloaded from the GEO database (https://www.ncbi.nlm.nih.gov/geo/).
