# Supplementary material for: Exploration of heterogeneity and recurrence signatures in hepatocellular carcinoma
Source: Mol Oncol. 2025 Feb 28;19(8):2388–411. doi: 10.1002/1878-0261.70012 (PMC12330942; doi:10.1002/1878-0261.70012)
Supplement: Supplementary file 1 — Fig. S1. ScRNA‐seq analysis in primary and relapsed HCC samples, related to Fig. 1. Fig. S2. Cell–cell communication analysis, related to Fig. 2. Fig. S3. Characteristics of tumor cells in relapsed and primary HCC, related to Fig. 3. Fig. S4. Heterogeneity of malignant cell transcriptome in RT and PT samples, related to Fig. 4. Fig. S5. Identification of potential gene modules associated with tumor cells by hdWGCNA. Fig. S6. Identification of key prognostic factors, related to Fig. 5. Fig. S7. Unsupervised consensus clustering analysis, related to Fig. 6. Fig. S8. Analysis of drug sensitivity, related to Fig. 7. [file MOL2-19-2388-s005.pdf]

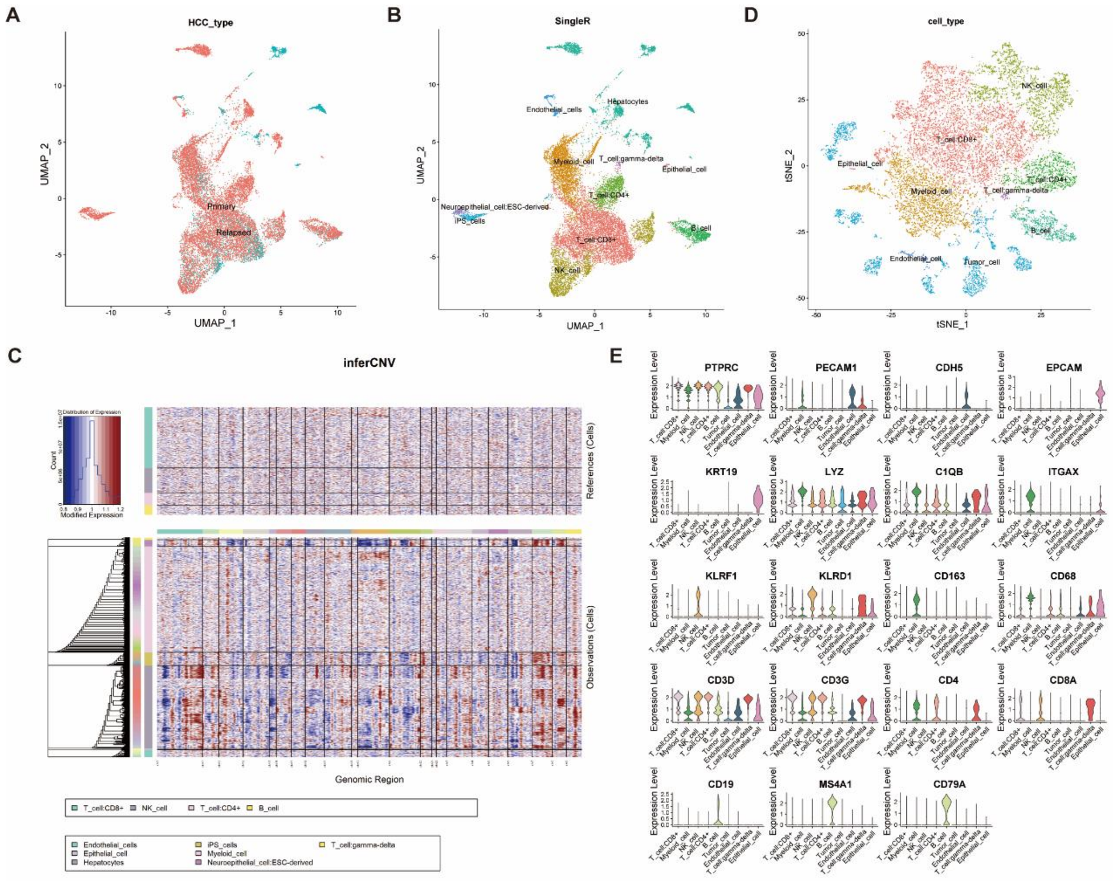

Figure S1. ScRNA-seq analysis in primary and relapsed HCC samples, related to Figure 1.

(A) The UMAP plot displaying cell origins by color, indicating PT or RT origin.

(B) UMAP plot of major cell types identified by SingleR package.

(C) Heatmap representing the CNV analysis, inferred from the single-cell RNA-seq data. All immune cells as the reference group

(D) TSNE plot of major cell types further identified by canonical markers.

(E) Violin plots showing the expression of marker genes for major cell types.

HCC, hepatocellular carcinoma; scRNA-seq, single-cell RNA sequencing; UMAP, uniform manifold approximation and projection; TSNE, t-distributed stochastic neighbor embedding; CNV, copy number variation; RT, relapsed tumor; PT, primary tumor.

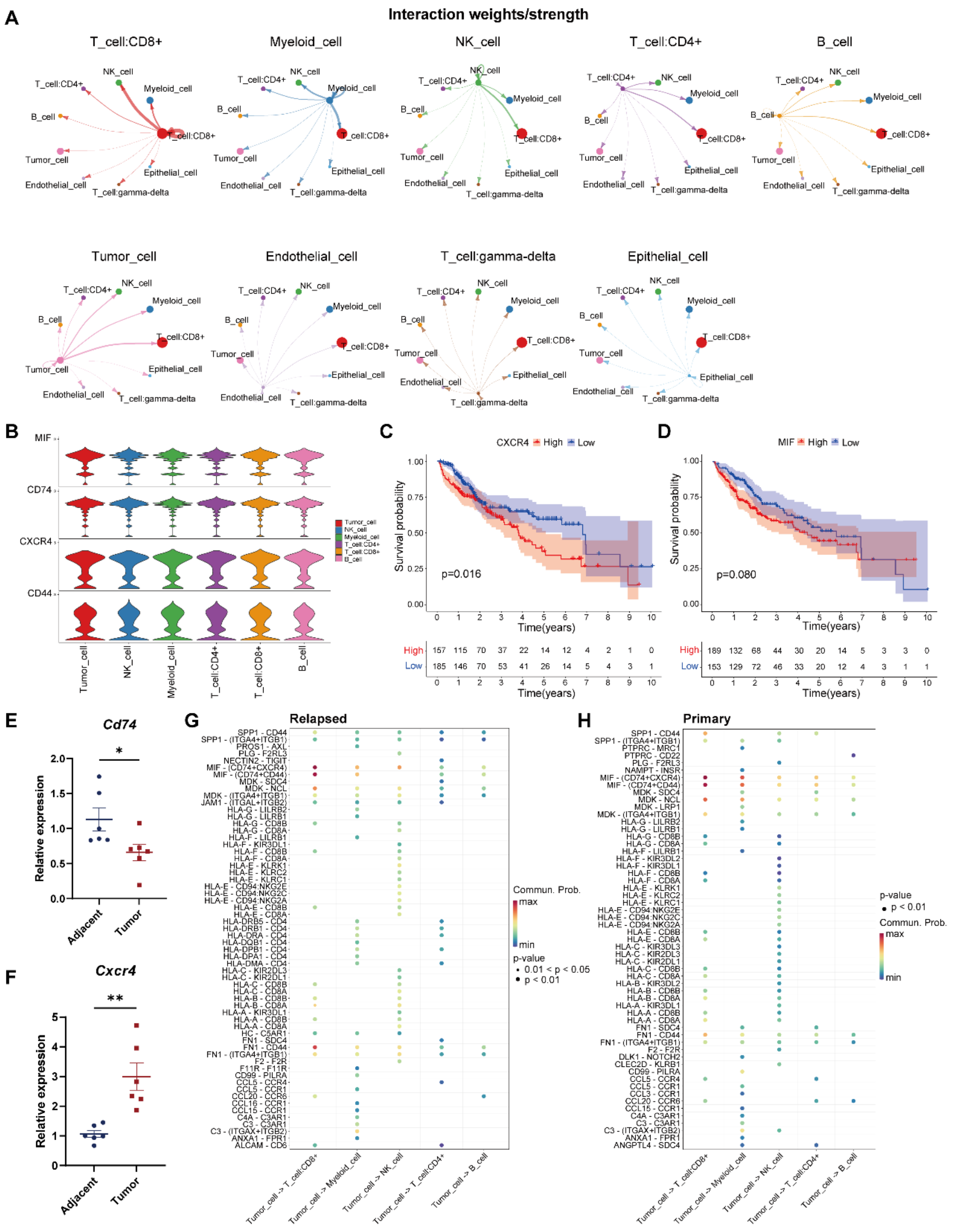

Figure S2. Cell-cell communication analysis, related to Figure 2.

(A) The signaling sent from each cell group for cell interactions.

(B) Violin plot showing the expression of related genes in the MIF signaling pathway.

(C and D) Kaplan-Meier analysis showing the overall survival rate of HCC patients, characterized by either low (blue) or high (red) expression of *CXCR4* and *MIF* (CXCR4: n = 170 samples for high group and n = 172 samples for low group; MIF: n = 189 samples for high group and n = 153 samples for low group). Significance was calculated by the log-rank test. The shaded areas around each curve indicate the 95% confidence intervals for the survival estimates.

(E and F) Differential mRNA expression levels of *Cd74* and *Cxcr4* genes in a murine hepatocellular carcinoma model (*n* = 6 mice). Relative mRNA levels were quantified by RT-qPCR with three technical replicates per sample. Data represent mean ± SEM (*n* = 6 biological replicates). Statistical significance between tumor and adjacent tissues were determined using paired two-tailed Student's *t* test (F) or Wilcoxon matched pairs signed rank test (E).

(G and H) Bubble plot showing interactions between tumor cells and major immune cells mediated by multiple ligand-receptor pairs or signaling pathways in relapsed and primary HCC tumor samples, respectively (*n* = 6 relapsed tumor samples; *n* = 12 primary tumor samples).

Statistical differences are denoted as **P* < 0.05, ***P* < 0.01. HCC, hepatocellular carcinoma; SEM, standard error of the mean; MIF, macrophage migration inhibitory factor.

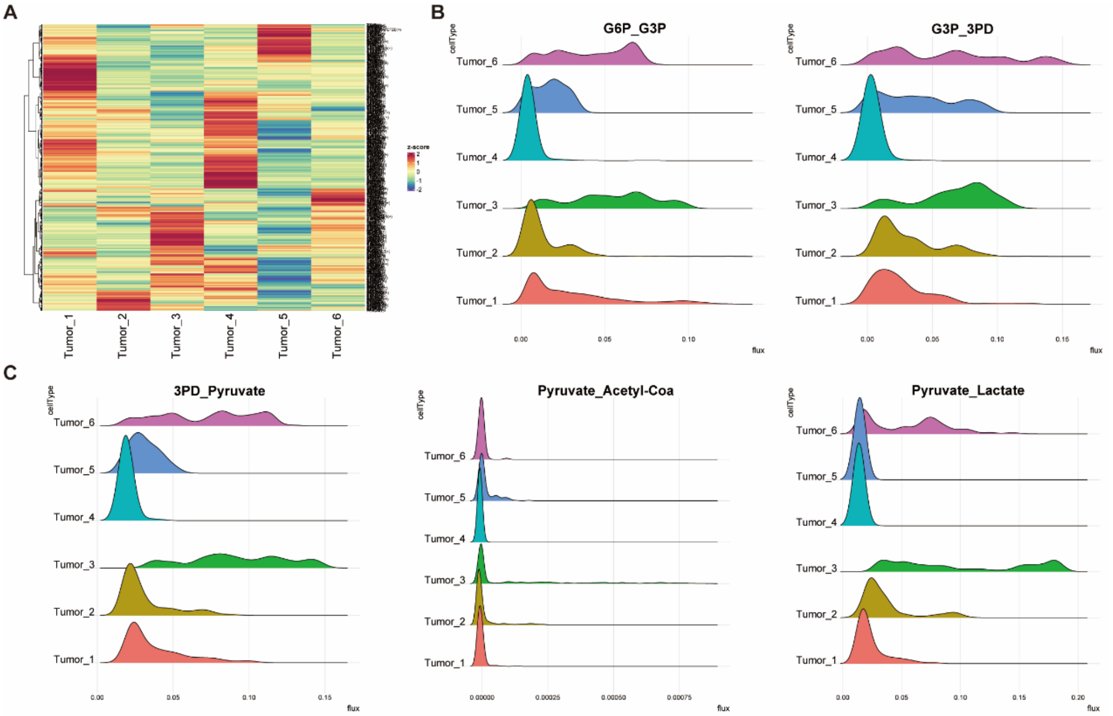

Figure S3. Characteristics of tumor cells in relapsed and primary HCC, related to Figure 3.

(A) Heatmap of the AUC scores of TF motifs estimated per tumor cell by SCENIC.

(B and C) Distribution of predicted fluxome of glycogen synthesis and TCA cycle modules in each tumor cell subgroup.

HCC, hepatocellular carcinoma; TF, transcription factor; AUC, the area under the curve; TCA, tricarboxylic acid.

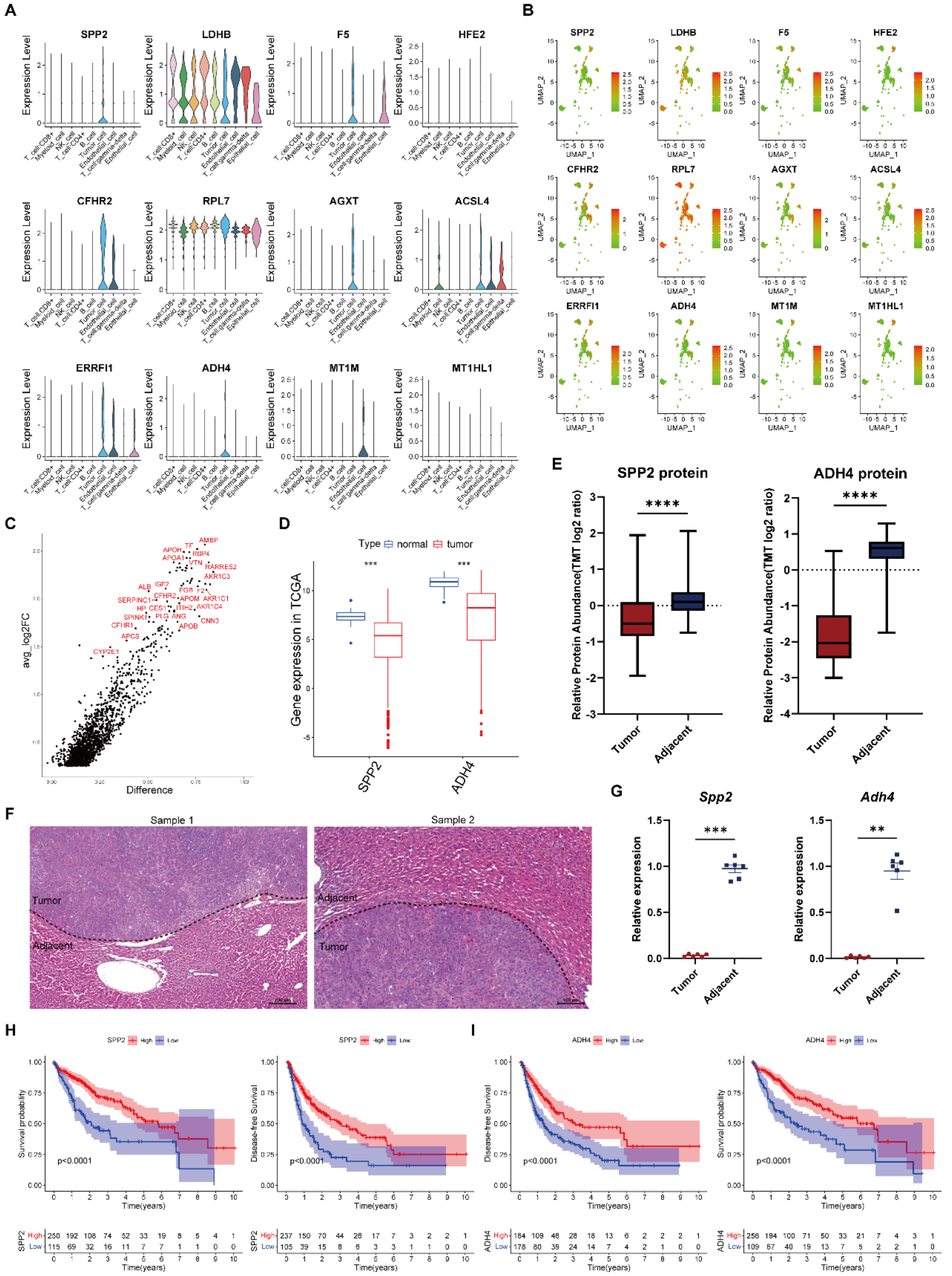

Figure S4. Heterogeneity of malignant cell transcriptome in RT and PT samples, related to Figure 4.

(A) Violin plot showing the expression of top12 DEGs between PT and RT malignant cells.

(B) UMAP plot showing the expression distribution of top12 differentially expressed genes with the most significant p-values between RT and PT malignant cells at the single-cell level.

(C) The volcano plot displaying significant DEGs in malignant cells.

(D) The boxplot showing the expression levels of the *SPP2* and *ADH4* genes in the TCGA dataset with normal samples (*n* = 50 samples) vs. tumor samples (*n* = 374 samples). Statistical differences were analyzed by unpaired two-tailed Wilcoxon rank-sum test. The box represents the IQR, with the central line indicating the median expression level. The whiskers extend to the minimum and maximum values within 1.5 times the IQR from the first and third quartiles, respectively. Outliers are represented by individual points beyond the whiskers.

(E) Protein levels of SPP2 and ADH4 in liver cancer were analyzed using the cProSite database (*n* = 159 pairs of tumor/adjacent samples collected from 159 HCC patients). The *P* values between groups were calculated by the unpaired two-tailed Wilcoxon rank-sum test. The box represents the IQR, with the central line indicating the median expression level. The whiskers extend to the minimum and maximum values within 1.5 times the IQR from the first and third quartiles, respectively.

(F) Representative images of HE staining in FFPE tissues, including tumor tissue and adjacent non-tumor tissue from a murine hepatocellular carcinoma model. Representative HE staining images from samples of three mice at 20× magnification; 200 μm or 100 μm scale bar (*n* = 6 slides, with 2 slides from samples of each mouse).

(G) RT-qPCR for *Spp2* and *Adh4* (*n* = 6 mice). Relative mRNA levels were quantified by RT-qPCR with three technical replicates per sample. Data represent mean ± SEM (*n* = 6 biological replicates). Statistical significance between tumor and adjacent tissues were determined using paired two-tailed Student's *t* test (left) or Wilcoxon matched pairs signed rank test (right).

(H) Survival curves for overall survival and disease-free survival in HCC patients stratified by high and low *SPP2* expression (*n* = 237 samples for high group and *n* = 105 samples for low group). Significance was calculated by the log-rank test. The shaded areas around each curve indicate the 95% confidence intervals for the survival estimates.

(I) Survival curves for overall survival and disease-free survival in HCC patients stratified by high and low *ADH4* expression (*n* = 164 samples for high group and *n* = 178 samples for low group). Significance was calculated by the log-rank test. The shaded areas around each curve indicate the 95% confidence intervals for the survival estimates.

Statistical differences are denoted as $^{**}P < 0.01$, $^{***}P < 0.001$, $^{****}P < 0.0001$. SEM, standard error of the mean. HCC, hepatocellular carcinoma; DEGs, differentially expressed genes; IQR, interquartile range; HE, Hematoxylin and Eosin; FFPE, Formalin-fixed paraffin-embedded; RT, relapsed tumor; PT, primary tumor.

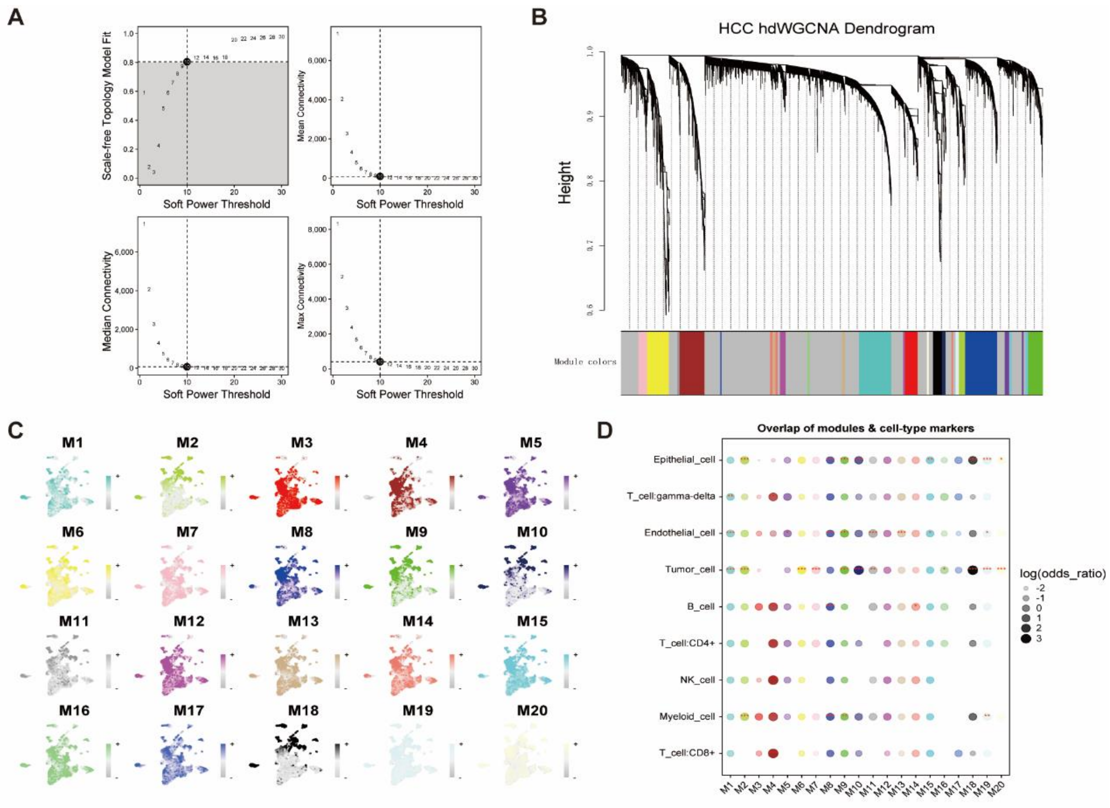

Figure S5. Identification of potential gene modules associated with tumor cells by hdWGCNA.

(A) The selection of soft-thresholding powers; left panels: the impact of soft-threshold power on the scale-free topology fit index; right panels: average network connectivity under different weighting coefficients.

(B) WGCNA dendrogram showing the different co-expression modules resulting from the network analysis.

(C) The UMAP plot displaying 20 modules colored by uniquely assigned colors for each module.

(D) Bubble plots of correlations between modules and cell types. The Pearson's correlation coefficient was employed to calculate the correlation.

WGCNA, weighted gene co-expression network analysis; UMAP, uniform manifold approximation and projection.

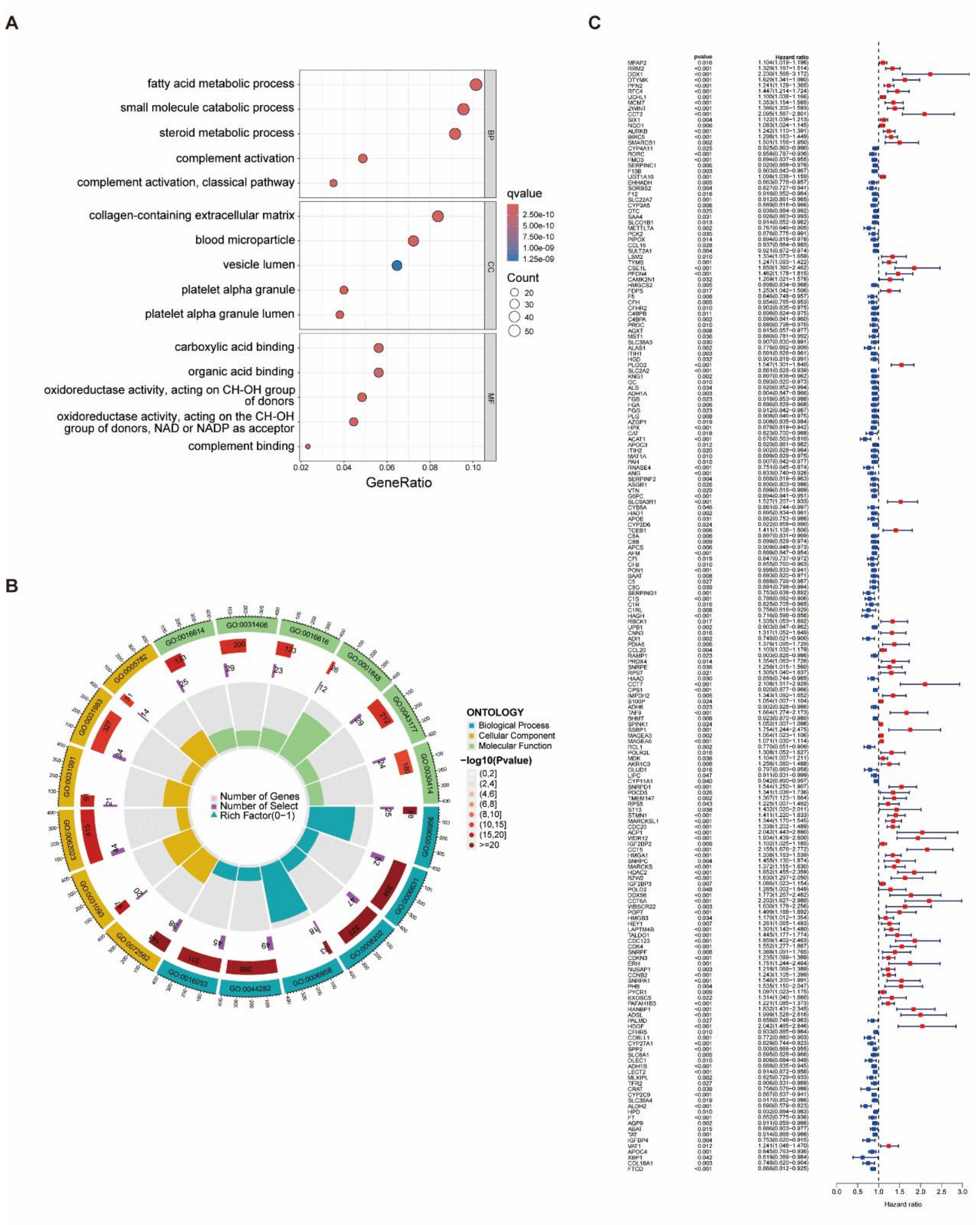

Figure S6. Identification of key prognostic factors, related to Figure 5.

(A and B) Bubble plot and circle plot of KEGG enrichment analysis for the 580 overlap genes.

(C) Univariate Cox analysis of the 580 overlap genes. A total of 195 potential prognostic genes were ultimately identified. The hazard ratio is presented as a point estimate, and the error bars represent the 95% confidence interval.

KEGG, Kyoto Encyclopedia of Genes and Genomes.

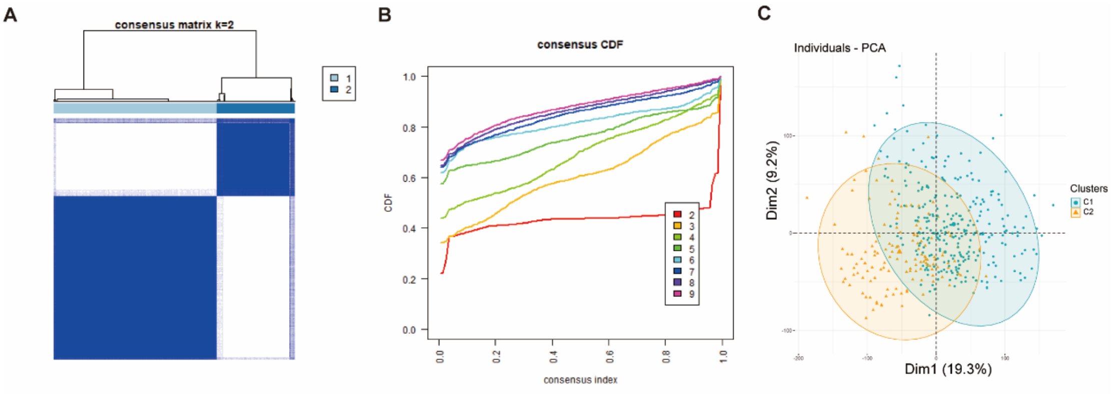

Figure S7. Unsupervised consensus clustering analysis, related to Figure 6.
(A) The consensus score matrix when k = 2.
(B) The CDF curves of consensus matrix for each k (indicated by colours).
(C) PCA diagram showing the distribution of two clusters.
CDF, cumulative distribution function; PCA, principal component analysis.

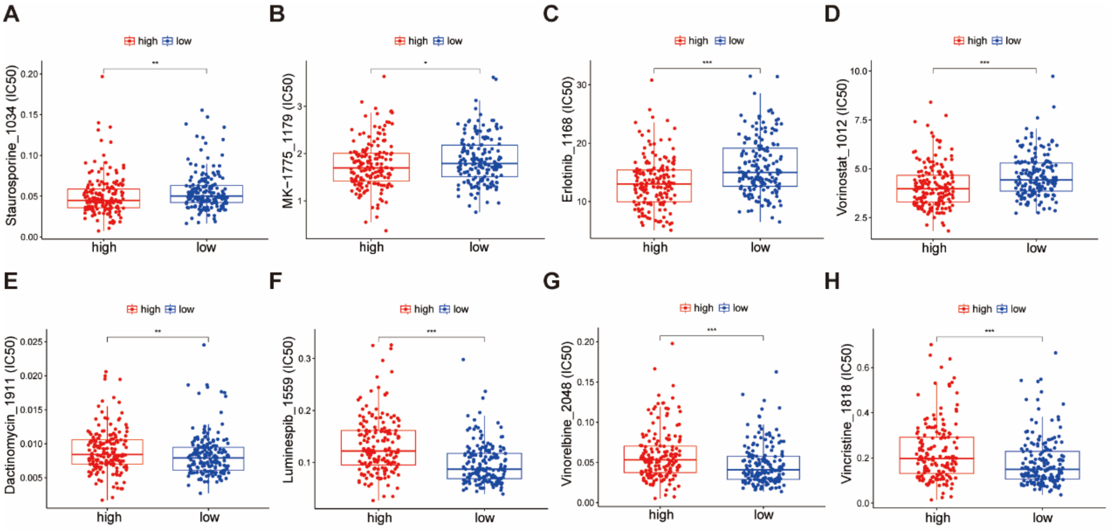

Figure S8. Analysis of drug sensitivity.
(A-H) Differences in chemotherapy drug sensitivity between two risk groups (n = 171 samples for the high-risk group and n = 172 samples for the low-risk group). The P values were measured using the unpaired two-tailed Wilcoxon rank-sum test. The box represents the IQR, with the central line indicating the median expression level. The whiskers extend to the minimum and maximum values within 1.5 times the IQR from the first and third quartiles, respectively.
Statistical differences are denoted as *P < 0.05, **P < 0.01, ***P < 0.001. IQR, interquartile range.
